# Celecoxib exerts protective effects in the vascular endothelium via COX-2-independent activation of AMPK-CREB-Nrf2 signalling

**DOI:** 10.1038/s41598-018-24548-z

**Published:** 2018-04-19

**Authors:** Fahad Al-Rashed, Damien Calay, Marie Lang, Clare C. Thornton, Andrea Bauer, Allan Kiprianos, Dorian O. Haskard, Anusha Seneviratne, Joseph J. Boyle, Alex H. Schönthal, Caroline P. Wheeler-Jones, Justin C. Mason

**Affiliations:** 1 Vascular Sciences and Rheumatology, Imperial Centre for Translational & Experimental Medicine, National Heart and Lung Institute, Imperial College London, London, UK; 2 King Fahad Cardiac Center, King Saud University, Riyadh, Saudi Arabia; 3 Department of Molecular Microbiology & Immunology, Keck School of Medicine, University of Southern California, Los Angeles, USA; 4 Comparative Biomedical Sciences, The Royal Veterinary College, London, UK

## Abstract

Although concern remains about the athero-thrombotic risk posed by cyclo-oxygenase (COX)-2-selective inhibitors, recent data implicates rofecoxib, while celecoxib appears equivalent to NSAIDs naproxen and ibuprofen. We investigated the hypothesis that celecoxib activates AMP kinase (AMPK) signalling to enhance vascular endothelial protection. In human arterial and venous endothelial cells (EC), and in contrast to ibuprofen and naproxen, celecoxib induced the protective protein heme oxygenase-1 (HO-1). Celecoxib derivative 2,5-dimethyl-celecoxib (DMC) which lacks COX-2 inhibition also upregulated HO-1, implicating a COX-2-independent mechanism. Celecoxib activated AMPKα^(Thr172)^ and CREB-1^(Ser133)^ phosphorylation leading to Nrf2 nuclear translocation. Importantly, these responses were not reproduced by ibuprofen or naproxen, while AMPKα silencing abrogated celecoxib-mediated CREB and Nrf2 activation. Moreover, celecoxib induced H-ferritin via the same pathway, and increased HO-1 and H-ferritin in the aortic endothelium of mice fed celecoxib (1000 ppm) or control chow. Functionally, celecoxib inhibited TNF-α-induced NF-κB p65^(Ser536)^ phosphorylation by activating AMPK. This attenuated VCAM-1 upregulation via induction of HO-1, a response reproduced by DMC but not ibuprofen or naproxen. Similarly, celecoxib prevented IL-1β-mediated induction of IL-6. Celecoxib enhances vascular protection via AMPK-CREB-Nrf2 signalling, a mechanism which may mitigate cardiovascular risk in patients prescribed celecoxib. Understanding NSAID heterogeneity and COX-2-independent signalling will ultimately lead to safer anti-inflammatory drugs.

## Introduction

The non-steroidal inflammatory drugs (NSAIDs), which include traditional non-selective NSAIDS (nsNSAIDs) and cyclo-oxygenase-2 selective NSAIDs (COXIBs), are widely prescribed and effective for symptom control in chronic diseases such as osteoarthritis (OA), ankylosing spondylitis and rheumatoid arthritis (RA). Although the principle cardiovascular and gastrointestinal side-effects are well recognised^1^, particular concern regarding COXIB-associated atherothrombotic risk persists^2^. Concern began with the publication of the APPROVE trial which reported increased thrombotic cardiovascular events in patients taking rofecoxib^3^. Rofecoxib was subsequently withdrawn and the concept of a COXIB class effect developed^2,4^. However, subsequent data has shown that there is considerable heterogeneity amongst nsNSAIDs and COXIBs, suggesting they should be considered as individual drugs rather than classes^5–7^. A recent meta-analysis investigated the most widely prescribed nsNSAIDs and COXIBs and their cardiovascular risk data^8^. The study confirmed the significant cardiovascular risk associated with rofecoxib. Importantly however, when rofecoxib data were removed from the COXIB group no difference in cardiovascular risk between COXIBs and nsNSAIDs remained, demonstrating skewing of the COXIB data by rofecoxib^8^. Furthermore, cardiovascular risk associated with celecoxib did not differ significantly from placebo. Celecoxib therapy conferred a lower risk of stroke and myocardial infarction than nsNSAIDs (other than naproxen)^8^, as previously shown in the CLASS trial^9^. The importance of the disease context and drug individuality is also revealed by studies of NSAID use in inflammatory arthritis where drug-associated cardiovascular risk is low, less than that seen in the control population, and principally associated with rofecoxib or diclofenac therapy^10,11^. Likewise, in patients with ankylosing spondylitis celecoxib therapy at an average daily dose of 300 mg was negatively associated with coronary artery disease^12^.

As a consequence of these observations, subsequent studies have compared the side-effects of individual NSAIDs in more detail. The risk of admission to hospital for heart failure was increased by etorocoxib, rofecoxib and seven nsNSAIDs but not by celecoxib at commonly used doses^13^. The Standard care versus Celecoxib Outcome Trial (SCOT) studied patients free of cardiovascular disease and compared cardiovascular safety in those prescribed continued nsNSAID therapy with those switched to celecoxib. The cardiovascular event rate was low (<1 per 100 patient years) and comparable between the two groups^14^. Finally, the recently reported ten year Prospective Randomized Evaluation of Celecoxib Integrated Safety versus Ibuprofen or Naproxen (PRECISION) trial enrolled 24,081 patients with RA or OA with established or significant risk of cardiovascular disease^15^. Although drug discontinuation rates were high and some caveats remain, the trial showed celecoxib to be noninferior to ibuprofen and naproxen with respect to cardiovascular risk, and to exhibit significantly improved gastrointestinal safety than either nsNSAID^15,16^.

The differences seen between individual nsNSAIDs and COXIBs, both within and between the two classes, led to the search for COX-2-independent actions of these drugs^17^. These have been identified in a variety of cell types. In vascular endothelial cells (EC) celecoxib inhibited TNF-α-mediated induction of tissue factor by minimising JNK mitogen-activated protein kinase (MAPK) activity, a response not seen with rofecoxib^18^. The growth inhibitory effect of celecoxib reflected inhibition of cyclin-dependent kinases^19^. Similarly, celecoxib but not rofecoxib increased heme oxygenase-1 (HO-1) expression and activity in human endothelium via changes in redox signalling^20^, a mechanism also identified in macrophages and vascular smooth muscle cells^21^.

These data led us to explore the role of COX-2 in celecoxib-mediated responses and to investigate COX-2-independent signalling in the vascular endothelium. We now report that celecoxib activates a novel anti-inflammatory AMPK-CREB-Nrf2-dependent pathway. This response was COX-2-independent and was not reproduced by the nsNSAIDs ibuprofen or naproxen. Activation of AMPK-CREB-Nrf2 signalling enhances EC protection and we propose that it plays a significant role in modifying cardiovascular risk.

## Results

### Celecoxib-mediated induction of HO-1 is COX-2-independent

**Figure 1:**
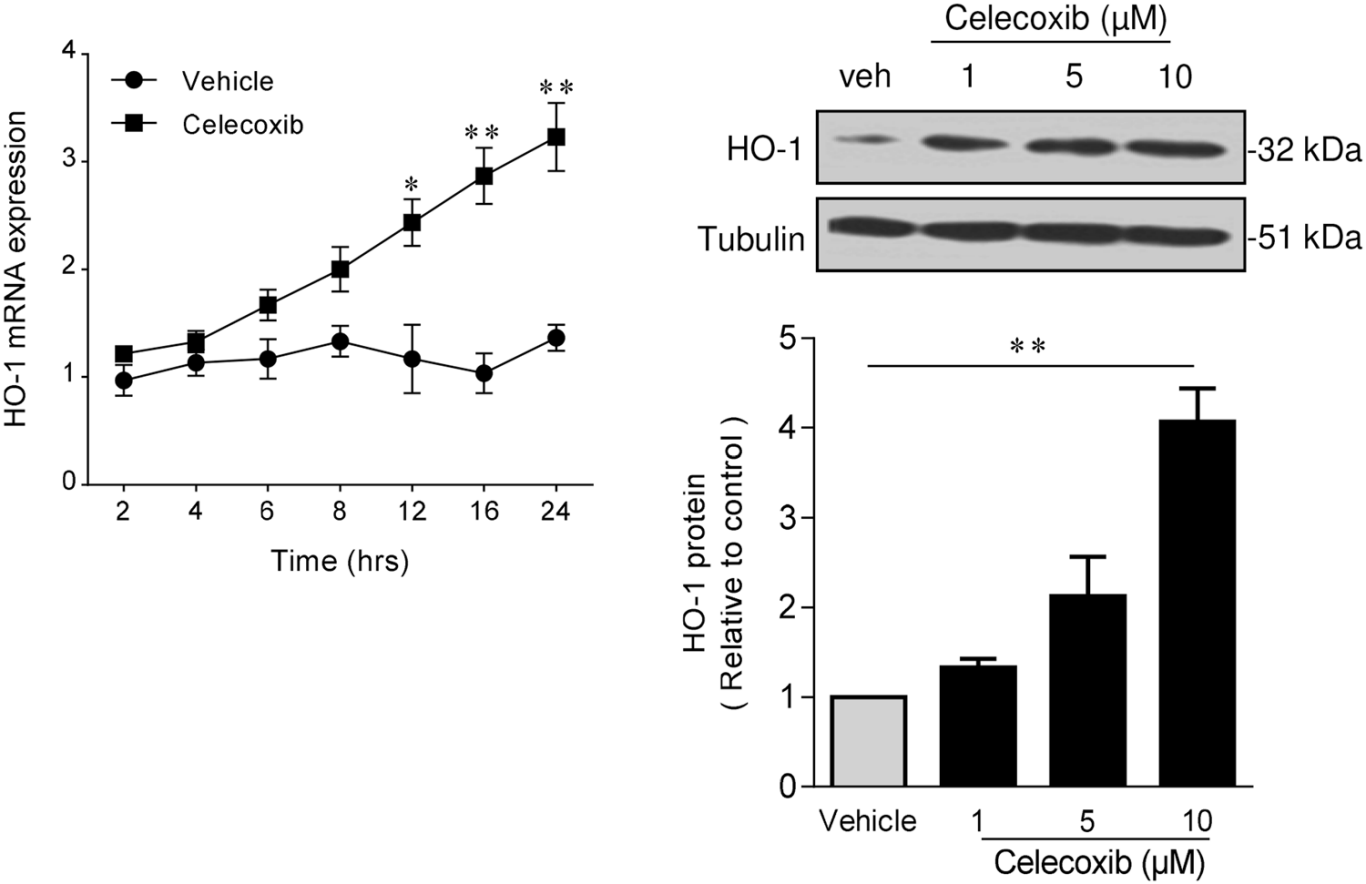
Celecoxib induces HO-1 in human endothelium. HUVECs were treated with celecoxib (up to 10 µM) or vehicle alone for up to 24 h, followed by: (**A**) RNA extraction and analysis of HO-1 by qRT-PCR, and (**B**) protein extraction and analysis by immunoblotting for HO-1 and α-tubulin. The histogram shows corresponding densitometry data corrected for α-tubulin. Data are expressed as the mean ± SEM (n = 4 experiments) and normalized to vehicle-treated cells, *P ≤ 0.05, **P ≤ 0.01, using a one-way ANOVA with a Bonferonni correction. Immunoblots shown have been cropped to conserve space, please see Supplementary file for original uncropped blots.

**Figure 2:**
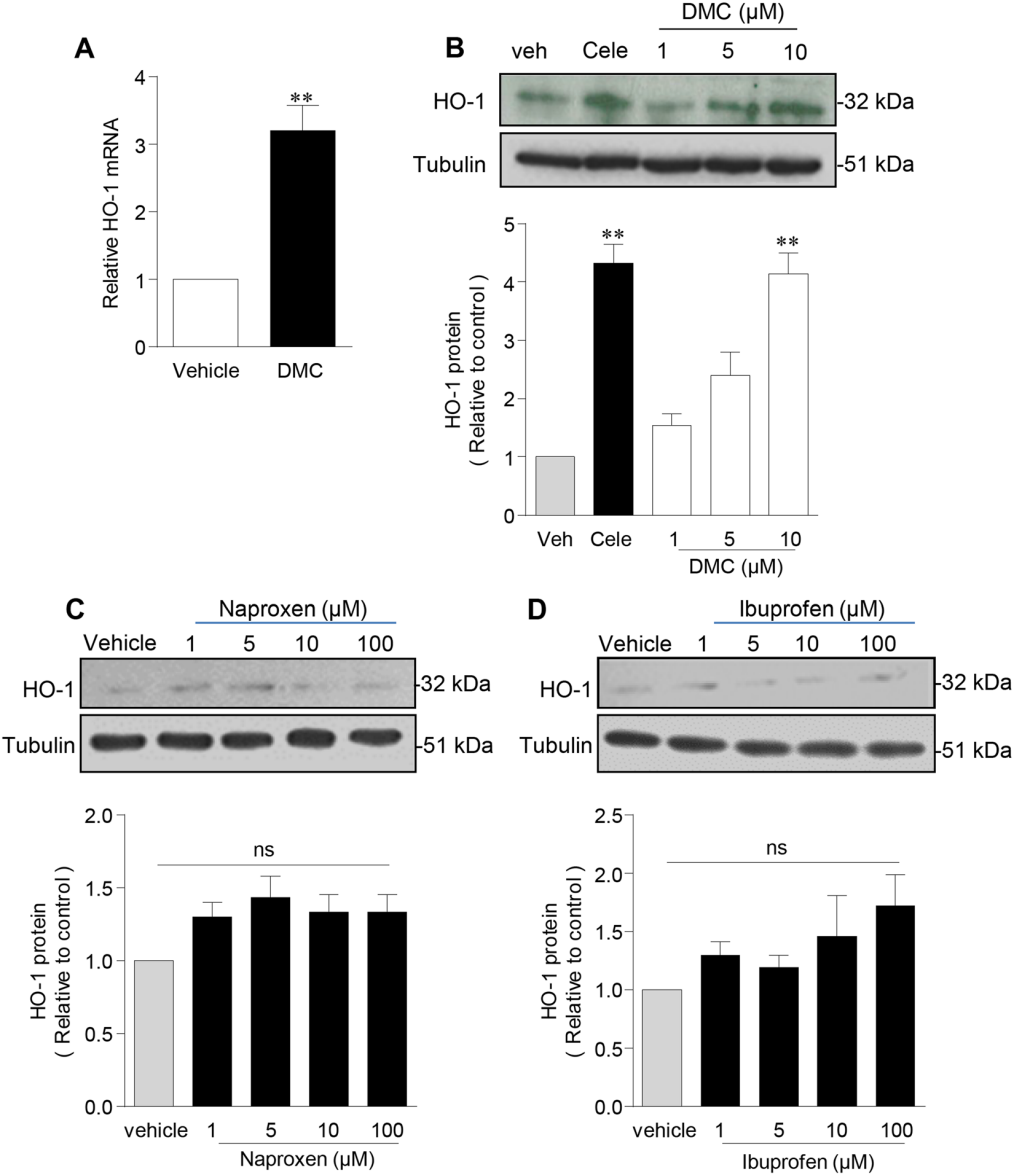
Celecoxib induction of HO-1 is COX-2-independent. HUVEC were treated with 2,5-Dimethyl-celecoxib (DMC) (up to 10 µM), celecoxib (10 µM) or vehicle alone for 24 h, followed by: (**A**) RNA extraction and analysis of HO-1 by qRT-PCR, and (**B**) protein extraction and analysis by immunoblotting for HO-1 and α-tubulin. (**C** and **D**) HUVEC were treated with vehicle alone or (**C**) naproxen, (**D**) ibuprofen (up to 100 µM) followed by immunoblotting analysis of HO-1. The histograms show corresponding densitometry data corrected for the α-tubulin and normalized to vehicle-treated cells. Data are expressed as the mean ± SEM (n = 4 experiments), *P ≤ 0.05, **P ≤ 0.01 using a one-sample t-test (**A**) and the one-way ANOVA with a Bonferonni correction (**B**–**D**). Immunoblots shown have been cropped to conserve space, please see Supplementary file for original uncropped blots.

Exposure of HUVEC to celecoxib resulted in the induction of HO-1, with a 3-fold increase in HO-1 mRNA seen after 16 and 24 h treatment, along with a corresponding concentration-dependent increase in protein (p < 0.01) (Fig. 1). This HO-1 response was used to interrogate the molecular mechanism underpinning vasculoprotective actions of celecoxib. Celecoxib derivative 2,5-dimethyl-celecoxib (DMC) was initially used to investigate the role of COX-2 inhibition. DMC is a structural analogue of celecoxib in which the 4-methylphenyl is replaced by a 2,5-dimethylphenyl, so preventing COX-2 inhibition^22^. The response of HUVEC to DMC mirrored that seen with celecoxib, suggesting a COX-2-independent mechanism. Thus, DMC induced a 3–4-fold increase in HO-1 mRNA and protein in a concentration-dependent manner (Fig. 2A,B). No HUVEC toxicity was seen with either drug at concentrations up to 10 μM, while as expected cell viability began to fall when the concentration of DMC was increased to 30 μM (Supplementary Figure 1A,B). Moreover, the presence of cycloheximide attenuated celecoxib-mediated induction of HO-1 (p < 0.001), confirming dependence upon *de novo* protein synthesis (Supplementary Figure 1C).

Naproxen and ibuprofen are two of the most widely prescribed nsNSAIDs. In contrast to celecoxib and DMC, they failed to induce HO-1 in HUVEC at concentrations up to 100 μM (Fig. 2C,D). Together these data suggest that concentrations of celecoxib and DMC up to 10 μM activate a downstream COX-2-independent signalling pathway that is not targeted by either naproxen or ibuprofen.

### Celecoxib-mediated activation of AMPKα induces endothelial HO-1

**Figure 3:**
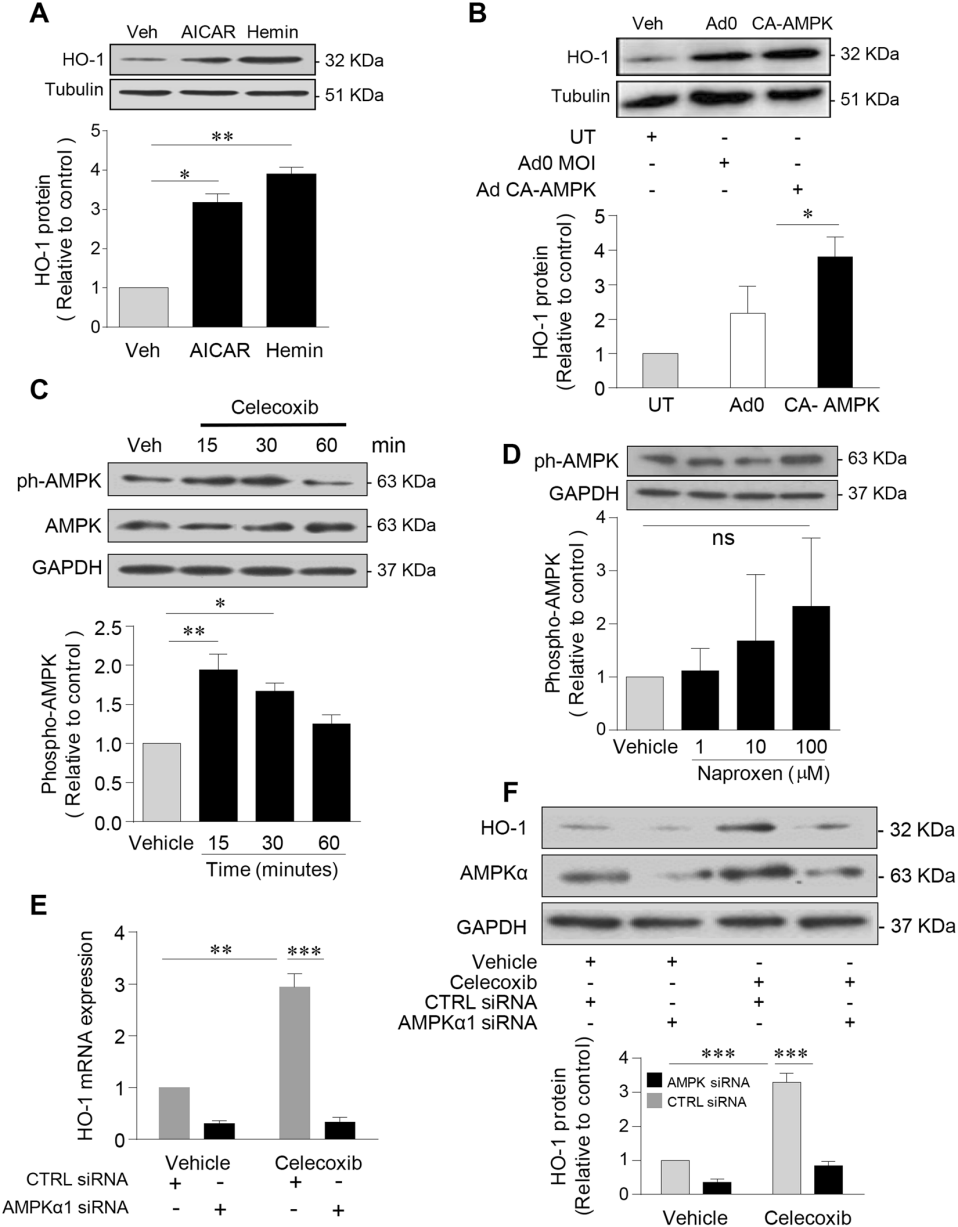
Celecoxib activates AMPK to induce HO-1. HUVECs were treated with: (**A**) vehicle alone, AICAR (1 mM for 24 h) or hemin (0.2 µM for 6 h, positive control), or (**B**) transfected with Ad0 (control virus), Ad-CA-AMPK (MOI 100 ifu/cell) or left untreated for 24 h, followed by protein extraction and immunoblotting for HO-1 or α-tubulin. (**C**) HUVECs were treated with celecoxib (10 μM) for up to 60 mins, or (**D**) naproxen up to 100 μM for 15 mins, prior to protein extraction and immunoblotting for phospho-AMPK^Thr172^ and GAPDH. (**E**,**F**) HUVECs were left untransfected or transfected with control siRNA (CTRL) or AMPKα1 siRNA (50 nM) and cultured for 48 h prior to treatment with vehicle or celecoxib (10 µM) for 24 h and (**E**) analysis of HO-1 by qRT-PCR, or (**F**) protein extraction and immunoblotting for HO-1, total-AMPK and GAPDH. Data are expressed as the mean ± SEM of at least 3 separate experiments. The histograms show corresponding densitometry data corrected for α-tubulin or GAPDH and normalized to vehicle-treated cells. *P ≤ 0.05, **P ≤ 0.01, **P ≤ 0.001, using the one-way ANOVA + Bonferonni correction or a two-way ANOVA. Immunoblots shown have been cropped to conserve space, please see Supplementary file for original uncropped blots.

Given that AMPK agonist AICAR increased endothelial HO-1 to a level approaching that seen in response to the positive control hemin (protoporphyrin IX) (Fig. 3A)^23^, and in light of our data linking AMPK activation to HO-1 expression in human EC^24^, we investigated AMPK as a celecoxib signalling intermediary. First, the 3–4-fold induction of HO-1 seen in response to AICAR was reproduced by transfection of HUVEC with an adenoviral vector expressing constitutively-active AMPK (Fig. 3A,B). Treatment of HUVEC with celecoxib for up to 60 mins led to a 2-fold increase in AMPKα activation, as evidenced by phosphorylation at Thr^172^ which was maximal after 15 mins (Fig. 3C). Of note, neither exposure to naproxen (Fig. 3D), nor treatment with ibuprofen (Supplementary Figure 2A) led to a significant increase in AMPKα phosphorylation.

Next, a loss of function approach using validated siRNA^24,25^ was adopted to demonstrate the role of AMPKα. Exposure of HUVEC to celecoxib for 24 h increased total AMPKα by 70%, while protein expression in untreated and celecoxib-treated cells was significantly reduced by AMPKα siRNA (p < 0.001, Supplementary Figure 2B). The induction of HO-1 mRNA by celecoxib in EC depleted of AMPKα was completely attenuated (p < 0.001, Fig. 3E). Similarly, the increase in HO-1 protein was inhibited by AMPKα silencing, while control siRNA had no significant effect (Fig. 3F).

### Celecoxib induction of HO-1 involves an AMPK-CREB-dependent pathway

**Figure 4:**
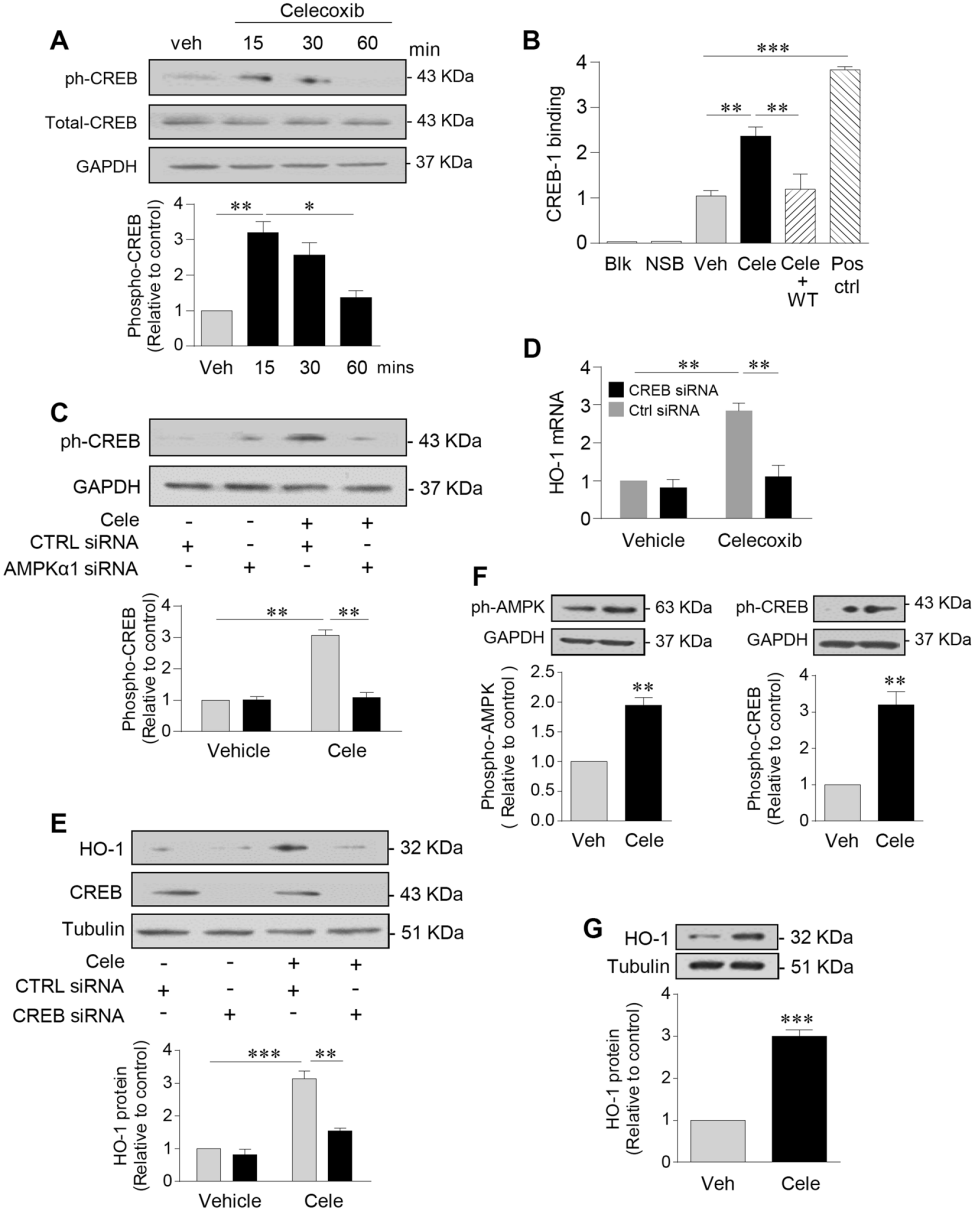
Celecoxib induces HO-1 in HUVEC and HAEC, by activating AMPK and CREB. (**A**) HUVECs were treated with vehicle (veh) or celecoxib (10 μM) for up to 60 mins, prior to lysis and immunoblotting for phospho-CREB^Ser133^, total CREB and GAPDH. (**B**) Nuclear lysates, isolated from HUVEC following treatment with celecoxib 10 μM (Cele) or vehicle control for 30 mins, were analysed using a phospho-CREB transcription factor assay kit. Unstimulated Hela cells were used as a positive control (CTRL) and a wild-type oligonucleotide sequence (WT) was used for competitive binding. CREB-binding is expressed relative to vehicle-treated cells. (**C**,**D**) HUVECs were left untransfected or transfected with control siRNA (CTRL) or CREB siRNA (50 nM) and cultured for 48 h prior to treatment with vehicle or celecoxib (10 µM) for (**C**) 15 mins followed by immunoblotting for phospho-CREB and GAPDH, or for 24 h followed by analysis of (**D**) HO-1 by qRT-PCR and (**E**) HO-1 protein by immunoblotting. (**F**) HAEC were treated with celecoxib (10 μM) or vehicle for 15 mins prior to immunoblotting for: phospho-AMPK^Thr172^, phospho-CREB^Ser133^ and GAPDH. (**G**) HAECs were treated with celecoxib (10 μM) or vehicle for 24 h prior to immunoblotting for HO-1. Data are expressed as the mean ± SEM of at least 3 separate experiments. The histograms show corresponding densitometry data corrected for α-tubulin or GAPDH and normalized to vehicle-treated cells. *P ≤ 0.05, **P ≤ 0.01, **P ≤ 0.001, using the one-way with a Bonferonni correction or two-way ANOVA (**A**–**E**) and a one-sample t-test. (**F**,**G**) Immunoblots shown have been cropped to conserve space, please see Supplementary file for original uncropped blots.

To investigate further the transcriptional pathway regulating HO-1 induction by celecoxib, cyclic AMP-response element binding protein (CREB) was identified as a potential candidate. CREB activity has been linked positively to vasculoprotection^26^ and CREB is a direct downstream target of AMPK^27,28^. Treatment of HUVEC with celecoxib resulted in a 2–3-fold increase in CREB^Ser133^ phosphorylation after 15–30 mins (Fig. 4A). A DNA-binding ELISA further demonstrated CREB activation, with a significant 2-fold increase in CREB DNA binding seen following celecoxib treatment and when compared to the vehicle control. The specificity of this response was demonstrated by its complete suppression in the presence of a wild-type competitive oligonucleotide corresponding to the CRE consensus sequence (p < 0.01, Fig. 4B).

Silencing AMPKα prior to celecoxib treatment prevented the 3-fold increase in CREB phosphorylation (p < 0.01), suggesting that AMPKα acts upstream of CREB in HUVEC (Fig. 4C). siRNA oligonucleotides were used to demonstrate the functional role of CREB. Celecoxib had no effect on total CREB, while CREB-specific siRNA depleted the protein by up to 90% (Supplementary Figure 2). Loss of CREB abrogated the induction of HO-1 mRNA and protein by celecoxib (p < 0.01, Fig. 4D,E), further supporting the presence of an AMPK-CREB-dependent pathway. Moving away from venous EC, this pathway was also activated in human arterial EC (HAEC) following treatment with celecoxib. A 2-fold increase in phospho-AMPK^Thr172^ and a 3-fold increase in phospho-CREB^Ser133^ were seen following 15 and 30 mins exposure respectively (Fig. 4F). Likewise, treatment of HAEC with celecoxib for 24 h increased HO-1 protein by up to 3-fold (p < 0.001, Fig. 4G).

### Nrf2 acts downstream of celecoxib in the vascular endothelium

**Figure 5:**
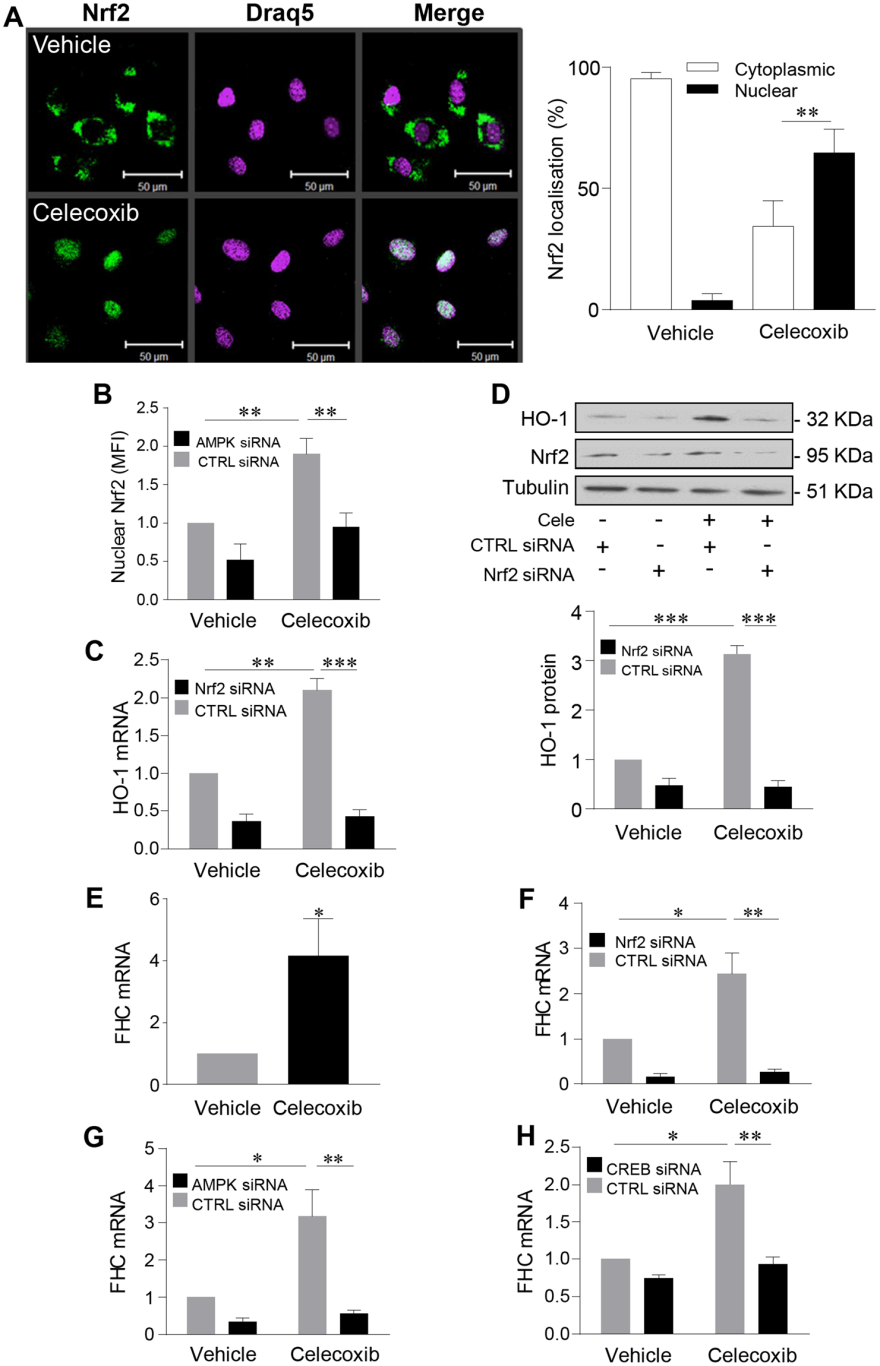
Celecoxib activates Nrf2 to induce HO-1 and H-ferritin. (**A**) HUVECs were left untreated or treated with celecoxib (10 μM) for 30 mins prior to fixation and staining with an anti-Nrf2 antibody and DRAQ-5 nuclear stain. Representative confocal images are shown along with a histogram representing pooled quantification data (n = 4). Data were analysed using Image J software and expressed as mean fluorescent intensity (MFI) of Nrf2 staining in the cytoplasm or nucleus. (**B**) HUVECs were transfected with control siRNA (CTRL) or AMPKα1 siRNA (50 nM) and cultured for 48 h prior to treatment with celecoxib and analysis of Nrf2 translocation as above. (**C**,**D**) HUVECs were transfected with control siRNA or pooled Nrf2 siRNAs (40 nM) and cultured for 48 h prior to celecoxib treatment (10 μM for 24 h). Changes in HO-1 were analysed by (**C**) qRT-PCR and (**D**) immunoblotting and the histogram shows corresponding densitometry data corrected for α-tubulin. (**E**) HUVECs were treated for 24 h with celecoxib or vehicle alone prior to analysis of H-ferritin (FHC) mRNA by qRT-PCR. (**F**–**H**) HUVECs were transfected with control siRNA or: (**F**) Nrf2 siRNA. (**G**) AMPKα1 siRNA, (**H**) CREB siRNA, for 48 h prior to treatment with vehicle or celecoxib and analysis of FHC mRNA by qRT-PCR. All data are derived from 4 independent experiments, normalized to vehicle-treated cells and expressed as the mean ± SEM. *P ≤ 0.05, **P ≤ 0.01, **P ≤ 0.001, using the two-way ANOVA or a one-sample t-test. (**E**) Immunoblots shown have been cropped to conserve space, please see Supplementary file for original uncropped blots.

Transcription factor nuclear factor (erythroid-derived 2)-like 2 (Nrf2) plays a central role in the regulation of HO-1^29^. Activation of Nrf2 is accompanied by its translocation to the nucleus and binding to anti-oxidant response elements (ARE) in target promoters. Nrf2 was confined to the cytoplasm in vehicle-treated HUVEC, while 64.8 ± 4.9% localised to the nucleus following treatment with celecoxib (Fig. 5A). Confocal imaging analysis of Nrf-2 nuclear fluorescence intensity confirmed that this response was AMPK-dependent, with AMPK silencing significantly attenuating Nrf2 nuclear translocation (p < 0.01, Fig. 5B). Nrf2 siRNA oligonucleotides were able to reduce HUVEC Nrf2 protein by up to 80% (Supplementary Figure 2D). Depletion of Nrf2 reduced basal HO-1 mRNA and protein levels and completely abrogated the celecoxib-induced increase in HO-1 seen in control siRNA-transfected cells (p < 0.01, Fig. 5C,D).

To explore further the downstream effect of Nrf2 activation by celecoxib, changes in the expression of four Nrf2 targets: H-ferritin, NAD(P)H dehydrogenase [quinone] 1 (NQO1); thioredoxin reductase 1 (Txnrd1) and thioredoxin were sought. Initial analyses demonstrated differential effects on these target genes with 4-fold and 1.5-fold increases in H-ferritin and Txnrd1 mRNA levels respectively following celecoxib treatment, and no change in either NQO1 or thioredoxin (Supplementary Figure 3A,B and Fig. 5E).

Subsequent experiments utilising control and specific siRNAs confirmed the role of Nrf2 in celecoxib-mediated induction of H-ferritin, with complete abrogation of the response following Nrf2 silencing (p < 0.01, Fig. 5F). Likewise dependence upon AMPK-CREB signalling was similarly demonstrated (p < 0.01, Fig. 5G,H). The role of AMPK, CREB (not shown) and Nrf2 was also confirmed in the more modest induction of Txnrd1 (Supplementary Figure 3C,D). Of note, Nrf2 activation alone is not sufficient to activate CREB, as sulforaphane treatment failed to induce CREB phosphorylation. In contrast, forskolin treatment, used as a positive control activated CREB (Supplementary Figure 3E).

### *In vivo* regulation of HO-1 and H-ferritin in response to celecoxib

To establish whether endothelial HO-1 and H-ferritin were altered by celecoxib dosing *in vivo*, C57Bl/6 mice were fed normal chow or a modified celecoxib-supplemented chow containing 1000 ppm celecoxib for 48 hours. Selection of the celecoxib concentration in the diet was based on previous studies demonstrating that the mean maximum plasma concentration achieved after a single dose of celecoxib in humans (1.53 µg/ml)^30^ can be achieved in mice receiving this diet^31,32^. Initial experiments confirmed the efficacy of this regimen by measuring serum prostaglandin E_2_ (PGE_2_) levels^33^. After 48 hours of celecoxib-treatment the PGE_2_ serum concentration fell from 107.7 ± 4.6 ng/ml to 60.4 ± 4.2 ng/ml when compared to control mice (p < 0.05, Supplementary Figure 4A).

**Figure 6:**
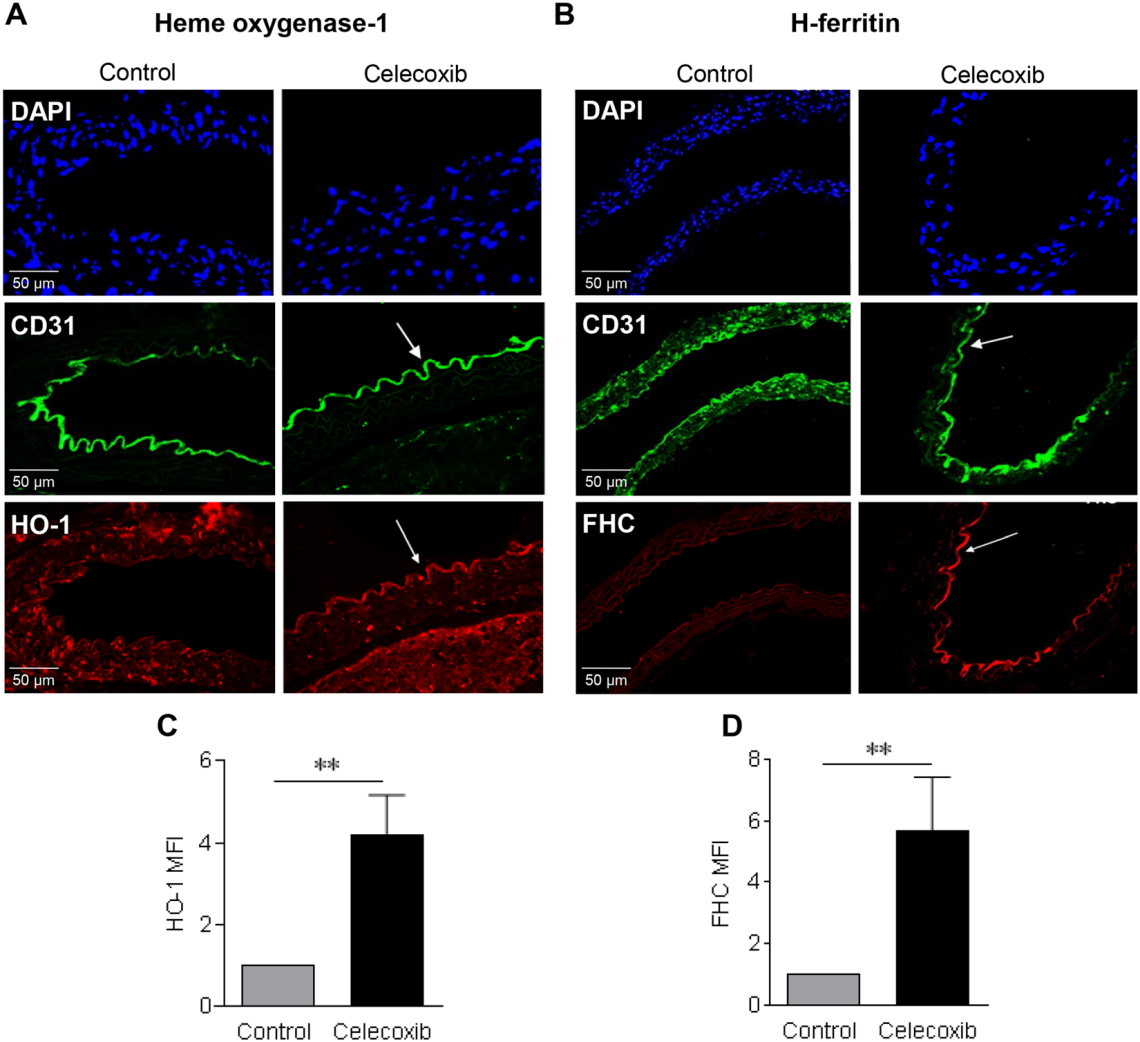
*In vivo* dosing of celecoxib upregulates aortic expression of HO-1 and H-ferritin. C57BL/6 female mice (n = 4 per group) were fed a diet containing 1000 ppm of celecoxib or matched standard laboratory diet for 48 h. Transverse sections of descending aorta were stained with: (**A**) an anti-HO-1 Ab or (**B**) an anti-H-ferritin (FHC) Ab. Anti-CD31 Ab was used to delineate endothelium and nuclei were visualized using DAPI nuclear dye. Representative confocal images (x40 magnification) of control and celecoxib-treated mice are shown. Histograms (**C**) HO-1 and (**D**) FHC show pooled quantification data from 4 independent experiments. Images were analysed by ImageJ software. Data are expressed as mean fluorescent intensity (MFI) of endothelial fluorescence ± SEM, representing MFI with antibody of interest in celecoxib or vehicle-treated mice divided by the MFI obtained with the control Ab and presented normalized to vehicle-treated animals. *P ≤ 0.05, **P ≤ 0.01, using an unpaired Student’s t-test. Images shown are at x40 magnification.

Transverse sections of the descending aorta were analysed using confocal immunofluorescence microscopy. In the control animals HO-1 was detectable in vascular smooth muscle cells, but not in the aortic endothelium of sequential sections in which the endothelium was identified by staining for CD31. In contrast, aortas from celecoxib-treated animals showed induction of HO-1 in the CD31^+^ endothelium lining the descending aorta, with an MFI 4-fold higher than untreated controls (p < 0.01, Fig. 6A). Similar experiments were performed using an antibody recognising H-ferritin. H-ferritin was barely detectable in the arterial wall of control mice aortas. However, in those animals exposed to celecoxib there was a 5–6-fold increase in the expression of H-ferritin in the vascular endothelium (p < 0.01, Fig. 6B).

### Celecoxib attenuates TNF-α-mediated signalling via AMPK activation and NF-κB inhibition

**Figure 7:**
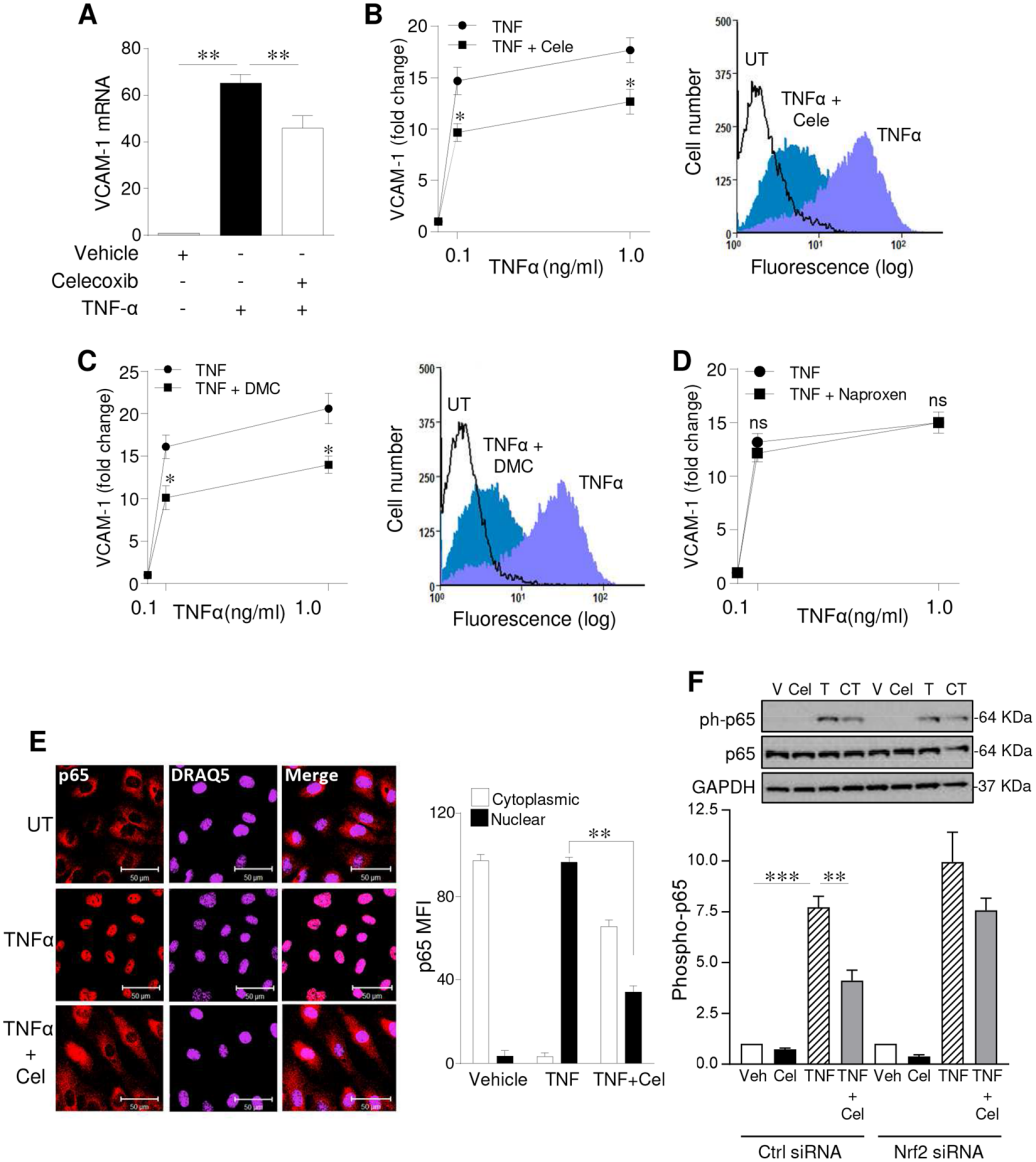
Celecoxib inhibits NF-κB activity to restrict VCAM-1 induction in vascular EC. HUVECs were pre-treated with vehicle, (**A**,**B**) celecoxib, (**C**) DMC or (**D**) naproxen (all 10 µM) for 24 h followed by TNF-α stimulation (up to 1 ng/ml) for (**A**) 6 h, with VCAM-1 analysed by qRT-PCR, and (**B**,**D**) 16 h followed by flow-cytometric analysis of VCAM-1, with data presented as relative fluorescence intensity (RFI), representing mean fluorescence intensity (MFI) with the VCAM-1 Ab divided by the MFI of the UT control (n = 4 experiments). (**E**,**F**) HUVECs were left untreated or treated with celecoxib for 24 h prior to addition of vehicle or TNF-α (0.1 ng/ml) for 30 minutes. (**E**) EC were fixed and stained with an anti-p65 antibody and DRAQ-5 nuclear stain. Representative confocal images are shown along with a histogram representing pooled quantification data (n = 3 experiments). Data were analysed using Image J software and expressed as the MFI of p65 staining in the cytoplasm or nucleus. (**F**) HUVECs were transfected with control siRNA (CTRL) or Nrf2 siRNA and cultured for 30 h prior to treatment with vehicle or celecoxib (10 µM) for 24 h and exposure to TNF-α (0.1 ng/ml) for 30 mins. Lysates were immunoblotted for phospho-p65^Ser536^, p65 and GAPDH. The fold change in phosphorylation was quantified using densitometry (n = 4 experiments), normalized with respect to GAPDH and expressed relative to the vehicle control. Data are expressed as the mean ± SEM, *P ≤ 0.05, **P ≤ 0.01, ***P ≤ 0.001, ns = non-significant using one-way or two-way ANOVA. Immunoblots shown have been cropped to conserve space, please see Supplementary file for original uncropped blots.

To investigate the COX-2-independent functional effects of celecoxib, HUVEC were treated with celecoxib or vehicle for 24 h prior to exposure to TNF-α for 6 h and analysis of VCAM-1 mRNA by qRT-PCR. The marked induction of VCAM-1 mRNA by TNF-α was reduced by up to 30% by celecoxib (p < 0.05, Fig. 7A). Similarly the 15-fold induction in VCAM-1 protein seen after treatment with 0.1 or 1.0 ng/ml TNF-α was significantly reduced but not completely abrogated by celecoxib (p < 0.05, Fig. 7B). Treatment with DMC reproduced these results, implying that this anti-inflammatory action of celecoxib was independent of COX-2 inhibition (Fig. 7C). Moreover, neither naproxen (Fig. 7D) nor ibuprofen (not shown) inhibited TNF-α-mediated induction of VCAM-1. Of note, celecoxib treatment predominantly affected VCAM-1, as similar experiments failed to show any significant inhibition of the TNF-α-induced upregulation of the cellular adhesion molecules E-selectin or ICAM-1 (Supplementary Figure 4B,C).

To investigate these responses further the impact of Nrf2 activity on the regulation of NF-κB p65 (RelA) was explored. As shown in Fig. 7E, TNF-α markedly increased the nuclear localisation of p65 from 3.5 ± 1.3% to 96.5 ± 1.2%, a response reduced to 34.3 ± 1.5% by celecoxib pre-treatment (p < 0.01). Nuclear translocation of p65 follows TNF-α-induced phosphorylation at Ser^536^, and celecoxib reduced this by up to 50% from 7.72 ± 0.5 to 4.11 ± 0.5-fold (p < 0.01, Fig. 7F). However, following siRNA silencing of Nrf2 the inhibition by celecoxib was attenuated, falling to 23.9% and no longer reached significance Fig. 7F.

**Figure 8:**
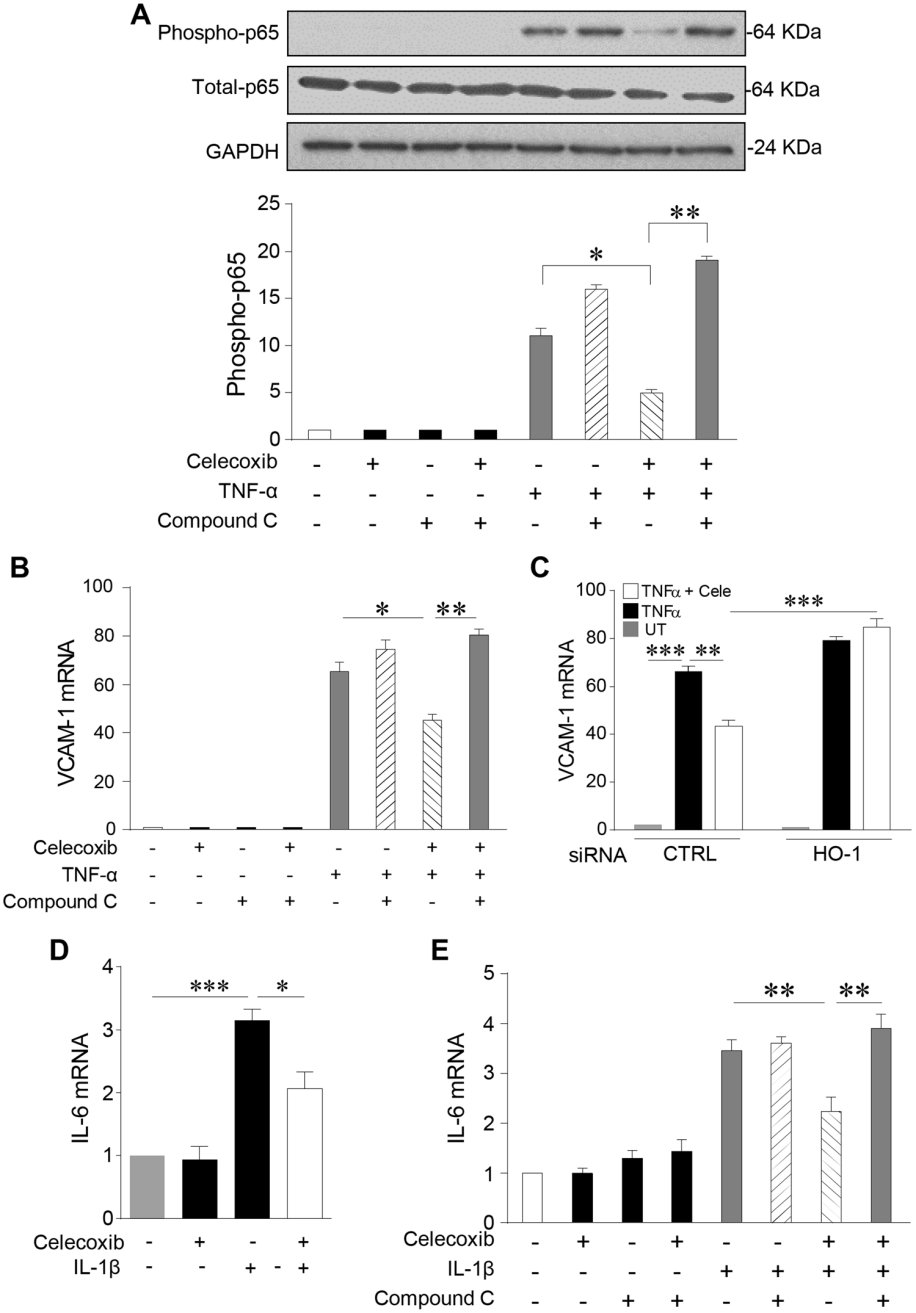
Celecoxib exerts anti-inflammatory effects via AMPK and induction of HO-1. HUVECs were pre-treated with Compound C (5 µM) or vehicle alone for 60 mins, prior to addition of celecoxib (10 μM) or vehicle for 24 h, followed by: (**A**) treatment with TNF-α (0.1 ng/ml) or vehicle for 30 mins and immunoblotting for p65, phospho-p65^Ser536^ and GAPDH. The histogram shows corresponding densitometry data for phospho-p65 corrected for GAPDH, or (**B**) treatment with TNF-α (0.1 ng/ml) or vehicle for 6 h, and analysis of VCAM-1 mRNA by qRT-PCR. (**C**) HUVECs were transfected with HO-1 or control (CTRL) siRNA (40 nM) for 48 h, prior to the addition of celecoxib or vehicle for 24 h and treatment with TNF-α (0.1 ng/ml) for 6 h. VCAM-1 mRNA was analysed by qRT-PCR. (**D**) HUVECs were left untreated or pre-treated with celecoxib for 24 h prior to addition of IL-1β (0.1 ng/ml) or vehicle for 4 h, and (**E**) HUVECs were left untreated or pre-treated with Compound C or vehicle alone for 60 mins, prior to addition of celecoxib for 24 h, followed by IL-1β (0.1 ng/ml) or vehicle for 4 h. Changes in IL-6 mRNA were analysed by qRT-PCR. Data in the figure are expressed as the mean ± SEM of 4 independent experiments and normalized to vehicle-treated cells. *P ≤ 0.05, **P ≤ 0.01, ***P ≤ 0.001, using one-way ANOVA with a Bonferonni correction. Immunoblots shown have been cropped to conserve space, please see Supplementary file for original uncropped blots.

In light of the demonstration that AMPK activity can interfere with NF-κB signalling^34^, its role in the anti-inflammatory actions of celecoxib was also explored. In these experiments Compound C proved to be the optimal means for inhibition of AMPK. Initial attempts using AMPK siRNA led to variable results between experiments. Dosing with Compound C (5 µM) was sufficient to significantly attenuate AMP phosphorylation and the protocol used reduced the duration of each experiment leading to improved consistency between experiments. TNF-α increased p65^Ser536^ phosphorylation by 11-fold and AMPK inhibition increased this by 30%. The presence of celecoxib significantly attenuated p65 phosphorylation, reducing it by 55%, a response that was completely reversed by Compound C (p < 0.01, Fig. 8A).

Turning to the functional consequences of AMPK-mediated inhibition of NF-κB, further experimentation suggested that activation of AMPK by celecoxib is required for its suppression of VCAM-1 induction by TNF-α. VCAM-1 upregulation was suppressed by celecoxib (TNF-α 74.4 ± 2.3- fold and TNF-α + celecoxib 45.3 ± 1.4-fold) and this change was reversed by Compound C (p < 0.01, Fig. 8B). Although these data are suggestive of a central role for AMPK, Compound C is not specific for AMPK and hence AMPK-independent actions cannot be completely excluded^35^.

Mechanistically, induction of HO-1 was responsible, at least in part, for the inhibition of VCAM-1 by celecoxib. The induction of VCAM-1 in control siRNA transfected cells was significantly reduced by celecoxib from 66.2 ± 2.2-fold to 43.4 ± 1.3-fold. However, when HO-1 was silenced the anti-inflammatory action of celecoxib was abrogated (Fig. 8C).

**Figure 9:**
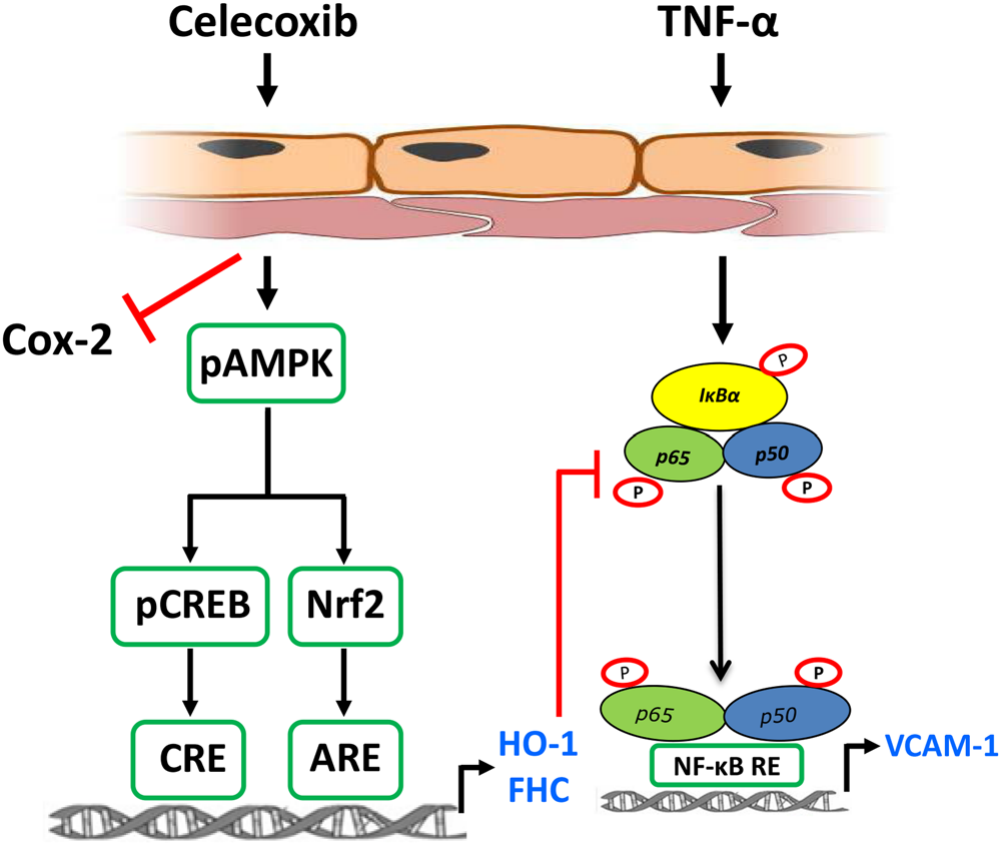
Summary of COX-2-independent AMPK-CREB-Nrf2 signalling pathway activated by celecoxib in the vascular endothelium. Treatment of human endothelial cells results in COX-2 independent signalling via phosphorylation of AMPK at threonine 172 (pAMPK). This leads to activation of CREB at serine 133 (pCREB) and nuclear translocation of Nrf2. The CREB and Nrf2 pathways are thought to act in parallel, binding to the CREB response element (CRE) and the antioxidant response element (ARE) respectively to upregulate the expression of the anti-oxidant, anti-inflammatory genes heme oxygenase-1 (HO-1) and H-Ferritin (FHC). The anti-inflammatory actions of this pathway include inhibition of TNF-α-mediated activation and nuclear translocation of p65. This response attenuated the pro-inflammatory upregulation of the cellular adhesion molecule vascular cell adhesion molecule-1 (VCAM-1).

To explore the anti-inflammatory actions of celecoxib in the endothelium more widely, the pro-inflammatory effect of IL-1β was explored, using induction of IL-6 as an end-point. Treatment of HUVEC with IL-1β resulted in a 3-fold increase in IL-6 mRNA. The presence of celecoxib was able to reduce this response by 33% from 3.20 ± 0.1 to 2.06 ± 0.14-fold (p < 0.05, Fig. 8D). Once again, inhibition of AMPK activity by Compound C completely reversed the anti-inflammatory action of celecoxib (IL-1β 3.5 ± 0.13-fold; IL-1β + celecoxib 2.2 ± 0.16-fold; and IL-1β+ celecoxib + Compound C 3.9 ± 0.17-fold) (p < 0.01, Fig. 8E). Thus, celecoxib activates an AMPK-CREB-Nrf2-dependent pathway which exerts COX-2-independent anti-inflammatory effects on the vascular endothelium through inhibition of TNF-α and IL-1β signalling (Fig. 9).

## Discussion

The ability of celecoxib to exert vasculoprotective effects^36,37^ may reflect in part anti-inflammatory, anti-oxidant actions upon macrophages and the vascular endothelium^20,21^. Activation of PI-3K/Akt, enhanced redox signalling and inhibition of JNK MAPK by celecoxib have been reported^18,20,21^. By comparing celecoxib with DMC and nsNSAIDS, we have now identified further COX-2-independent downstream targets of celecoxib within human vascular EC. Celecoxib activates AMPK-CREB-Nrf2-dependent signalling to enhance vascular protection in the endothelium via induction of HO-1, H-ferritin and Txnrd1. Single dosing of celecoxib in humans achieves a plasma cMax of approximately 2–8 μM^30,38^. Optimal *in vitro* responses were seen with 10 μM celecoxib, and this may reflect in part the extensive protein binding properties of the drug^31^. To pursue these observations’ an *in vivo* dosing regimen was chosen that achieves plasma concentrations in mice equivalent to those seen in humans^31,32^. *In vivo* dosing of celecoxib increased EC expression of both HO-1 and H-ferritin. The novel COX-2-independent pathway played a role in the anti-inflammatory actions of celecoxib including suppression of VCAM-1 upregulation by TNF-α, and IL-1β-mediated induction of IL-6.

Concern remains about the atherothrombotic risk associated with the clinical use of anti-inflammatory drugs and particularly the COXIBs^2^. However, nsNSAIDs and COXIBs should be considered as individual agents rather than as drug classes. The recently reported SCOT and PRECISION trials found no increased cardiovascular risk when comparing celecoxib with nsNSAIDs including naproxen and ibuprofen^14,15^, while the latest large meta-analysis suggests that the inclusion of rofecoxib has skewed the COXIB cardiovascular data^8^.

We investigated the hypothesis that the differences between the cardiovascular effects of individual nsNSAIDs and COXIBs reflect their COX-2-independent actions. The starting point was our observation that while celecoxib can induce the expression of HO-1 in human endothelium, this response was not seen with rofecoxib^20^. This action of celecoxib allowed direct comparison with DMC, a structural analogue of celecoxib generated to lack COX-2 inhibition^22^. These initial experiments (Figs 1 and 2) revealed directly comparable results between celecoxib and DMC in terms of HO-1 induction, so confirming the COX-2-independent nature of the response. Furthermore, neither naproxen nor ibuprofen was able to reproduce HO-1 induction.

An emerging relationship between AMPK and Nrf2 led us to explore this pathway in endothelial responses to celecoxib. AICAR, berberine and xanthohumol treatment of HUVEC, macrophages or fibroblasts leads to AMPK-dependent induction of HO-1 via activation of the transcription factor Nrf2^23,39,40^. Moreover, potent anti-inflammatory actions were observed in LPS-treated mice following activation of this pathway by berberine^39^. Celecoxib and DMC treatment of EC led to AMPK activation as evidenced by its phosphorylation at Thr^172^. In contrast, naproxen and ibuprofen failed to activate AMPK. Subsequent loss of function experiments revealed the AMPK-dependence of celecoxib-mediated HO-1 induction, Nrf2 activation and nuclear translocation.

AMPK, a heterotrimeric complex that acts as a critical metabolic regulator capable of responding to cellular stress^41^, plays an important role in endothelial metabolism and homeostasis^42^. Reduced AMPK activity is associated with oxidative stress and endothelial dysfunction^28,42^, while selective activation of AMPK in the vascular endothelium protects against the harmful effects associated with diabetes mellitus through induction of HO-1^43^. Thus, the finding that celecoxib is able to activate both AMPK and Nrf2 brings together redox and metabolic signalling, a combination that may help to minimise cardiovascular risk in patients.

CREB was constitutively expressed in both HUVEC and HAEC and activated by celecoxib as evidenced by phosphorylation of Ser^133^ in its kinase-inducible domain and increased binding to a CRE consensus sequence. Silencing of CREB attenuated HO-1 induction by celecoxib. CREB is a direct target of AMPK and its activation is considered vasculoprotective. CREB contributes to the regulation of cell metabolism, differentiation and survival^44^, and targeted inhibition in the heart predisposes to mitochondrial dysfunction, leading to oxidative stress and increased mortality^45^. Early aortic depletion of CREB is reported in rodent models of vascular disease^26^.

The combined data suggest that celecoxib has the potential to impart vasculoprotective effects via a COX-2-independent molecular mechanism comprising 3 signalling components, all of which have their own cytoprotective actions. Initial investigation of the broader downstream effects of the AMPK-CREB-Nrf2 pathway focused on additional Nrf2 targets. Selective effects were seen with H-ferritin, Txnrd1 and HO-1 induced, while there was no change in either NQO1 or thioredoxin. This observation may reflect cell-specific effects and/or competition for Nrf2 binding at individual promoters. The most marked response to celecoxib was seen with HO-1 and H-ferritin and this was reproduced *in vivo*, with a 4-6-fold increase in endothelial expression seen in the murine aorta.

AMPK-Nrf2 signalling has been linked to the regulation of H-ferritin in T lymphocytes^46^. H-ferritin is widely expressed and plays a central role in the handling and storage of intracellular iron^47^. H-ferritin acts as a catalyst for the conversion of Fe^2+^ to Fe^3+^ and, along with L-ferritin, exerts an anti-oxidant action by removing excess free iron. A close functional relationship exists between H-ferritin and HO-1, including evidence suggesting that H-ferritin may on occasion mediate the protective effect of HO-1 against oxidative stress^48^.

The induction of VCAM-1 by TNF-α was used to initially investigate the functional effects of the celecoxib-activated AMPK-CREB-Nrf2 pathway. Pre-treatment with celecoxib or DMC significantly inhibited the induction of VCAM-1. However, neither naproxen nor ibuprofen was able to reproduce this effect. Celecoxib attenuated both phosphorylation and nuclear translocation of p65 in response to TNF-α. Experiments utilizing Compound C suggested that the inhibition of NF-κB signalling and IL-1β-mediated induction of IL-6 by celecoxib required activation of AMPK.

The outcome of the interaction between AMPK and NF-κB signalling appears to be context dependent. Thus, following activation by celecoxib, AMPK attenuated endothelial NF-κB activation by pro-inflammatory cytokines. Metformin is also reported to inhibit TNF-α-mediated activation of NF-κB via AMPK, while similarly AMPK activity attenuates fatty acid-induced p65 translocation^49,50^. In contrast, AMPK activity induced following oxygen and glucose deprivation enhanced NF-κB signalling and protected endothelial cells against apoptosis, most likely via the induction of Bcl-2 and survivin^51^. Thus, activation of AMPK may modulate NF-κB signalling by switching the binding of p65 and/or recruitment of its co-activators away from pro-inflammatory targets to the promoters of anti-inflammatory, anti-apoptotic and anti-oxidant genes. Although the mechanism(s) underpinning such responses remains to be established, HO-1 may contribute. The reduction in VCAM-1 upregulation seen in EC pre-treated with celecoxib and exposed to TNF-α was reversed by HO-1 silencing. Moreover, HO-1 has been reported to inhibit TNF-α-induced activation of NF-κB and adhesion molecule upregulation in EC^52,53^.

CREB and Nrf2 are members of the bZip family and we have previously suggested that transcriptional co-operation might occur between CREB1 and Nrf2 to optimise HO-1 induction^54^. Similarly heterodimerization between Nrf2 and another bZip family member ATF4, facilitates the induction of HO-1 by cadmium chloride^55^. Further experiments sought to investigate whether Nrf2 and CREB were acting in combination. Direct activation of Nrf2 in HUVECs with sulforaphane failed to phosphorylate CREB, suggesting that following activation, Nrf2 and CREB were acting in parallel (Fig. 9). Silencing of Nrf2 partially inhibited celecoxib-mediated inhibition of TNF-α-induced p65 phosphorylation. We propose that this is likely, at least in part, to be related to the reduction in HO-1 seen in Nrf2-depleted cells. Thus, following celecoxib treatment, AMPK phosphorylation leads to activation of CREB and Nrf2-dependent pathways that may act in parallel to induce anti-inflammatory genes including HO-1 and H-ferritin. Of note, a cAMP/PKA/CREB pathway has been implicated in inhibition of p65 translocation following treatment with phosphodiesterase antagonists^56^, while in macrophages Nrf2 specifically inhibits inflammation-induced transcription mediated by p65^57^.

## Conclusion

In contrast to ibuprofen and naproxen, celecoxib activates COX-2-independent AMPK-CREB-Nrf2 pathways to drive HO-1, H-ferritin and Txnrd1 expression in human EC. This response results in significant anti-inflammatory effects including inhibition of TNF-α-mediated NF-κB signalling, and IL-1β induction of IL-6. We propose that this and other COX-2-independent actions help to minimise adverse effects in the vasculature and contribute to the observation that celecoxib improves endothelial function in those with coronary artery disease^36^ and hypertension^37^. Recent data suggests that the cardiovascular risk associated with standard dose celecoxib is not inferior to nsNSAIDS and that overall risk in patients with arthritis is very low, even in those with ischaemic heart disease^8,10,11,14,15^. In light of this, guidelines should now refocus attention of physicians to consideration of the relative risk of both cardiovascular and gastrointestinal side-effects for each patient when prescribing NSAIDs. Additional mechanistic data concerning the vasculoprotective actions of individual drugs including celecoxib is required and will ultimately lead to development of safer and more effective anti-inflammatory therapies.

## Materials and Methods

The datasets generated during and/or analysed during the current study are available from the corresponding author on reasonable request.

### Reagents and antibodies

Monoclonal antibodies (mAb) against HO-1 and phospho-p65^Ser536^ were from Abcam (Cambridge, UK). E-selectin mAb 1.2B6, ICAM-1 mAb 6.5B5 and VCAM-1 mAb 1.4C3 were generated in house. CD31 mAb P2B1 was generated in house from a clone purchased from Developmental Studies Hybridoma Bank, University of Iowa, Iowa City, IA. The following antibodies were purchased from Cell Signaling (Danvers, MA) AMPKα, phospho-AMPKα^Thr172^, CREB, phospho-CREB^Ser133^. Antibodies against p65 and Nrf2 were from Santa Cruz Biotechnology (Heidelberg, Germany), α-tubulin Sigma-Aldrich (Poole, UK) and GAPDH Millipore (Watford, UK). 2,5-Dimethyl-celecoxib was synthesized as described^58^. Celecoxib was purchased from Toronto Research Chemicals (Ontario, Canada), naproxen and ibuprofen from Tocris Bioscience (Bristol, UK). The concentrations of all three drugs used *in vitro* (celecoxib 1–10 µM, naproxen and ibuprofen 1–100 μM) included concentrations that can be achieved therapeutically in humans^30,38,59,60^. 5-Aminoimidazole-4-carboxamide ribonucleotide (AICAR) and Compound C were from Millipore, hemin and sulforaphane from Sigma-Aldrich, TNFα and IL-1β from R and D Systems (Abingdon, UK).

### Endothelial cells

Use of human EC was approved by Hammersmith Hospitals Research Ethics Committee (ref no. 06/Q0406/21). Informed consent was obtained and all experiments were performed in accordance with relevant guidelines and regulations. Human umbilical vein endothelial cells (HUVEC) were isolated by collagenase Type II digestion. Human aortic EC (HAEC) were purchased from Promocell, (Heidelberg, Germany). HUVEC and HAEC were propagated in M199 medium supplemented with 2 mM L-glutamine, 100 U/ml penicillin/streptomycin, 20% FCS, and 30 μg/ml heparinized endothelial cell growth factor (ECGF) (all from Sigma-Aldrich). Experiments were performed with passage 3–5 EC from at least three separate isolates, plated in M199/2 mM L-glutamine, 100 U/ml penicillin/streptomycin, 10% FCS, and 7.5 μg/ml ECGF. Pharmacological antagonists were added to the endothelial culture medium 30–60 min before celecoxib.

### Cell viability assay

HUVECs monolayers were treated with celecoxib, DMC or vehicle for 24 h. Cell viability was analysed by incubation with 20% 3-(4,5 dimethylthiazol-2-yl)-2,5-diphenyltetrazolium bromide (MTT) in a growth factor-free medium for 3 h. Plates were read on a Synergy HT plate reader and the number of viable cells determined by measuring the absorbance at 490 nm.

### siRNA Transfection

GeneFECTOR (3:50) (VennNova, Parkland Fl) and siRNA (40 nM final) were diluted in Opti-MEM I (Invitrogen, Paisley, UK) and equal volumes of each were mixed and incubated at r/t for 5 min. siRNA complexes were added drop wise to HUVECs cultured in Opti-MEM I medium. After incubation for 4 h, culture medium was replaced with EGM2 medium (Lonza, Wokingham, UK) overnight and subsequently by M199/10% FCS. The following validated siRNA oligonucleotides were employed^24,54,61^.

HO-1 (siHO-1 seq. 2) 5′- AACAUUGCCAGUGCCACCAAG-3′ (Qiagen, Manchester, UK).

AMPKα1 siRNA (Hs_PRKAA1_5) 5′-CCCACGATATTCTGTACA CAA-3′ (Qiagen).

CREB-1 pooled sequences (Dharmacon, Epsom, UK):

5′-GAGAGAGGTCCGTCTAATG-3′; 5′-CGTACAAACATACCAGATT-3′;

5′-GAGTGGAGATGCAGCTGTA-3′; 5′-TGACTTATCTTCTGATGCA-3′.

Nrf2 pooled sequences (Dharmacon):

5′-TGACAGAAGTTGACAATTA-3′; 5′-TAAAGTGGCTGCTCAGAAT-3′

5′-CCAAAGAGCAGTTCAATGA-3′; 5′-GAGAAAGAATTGCCTGTAA-3′.

The si-GENOME Non-Targeting siRNA #1 from Dharmacon containing 4 mismatches to any human, mouse or rat gene was used as a negative control.

### Quantitative real-time PCR

Quantitative real-time PCR (qRT-PCR) was performed using the CFX96 Real-Time system and C1000 Thermal cycler (Bio-Rad, Hercules, CA), with data calculated in relation to housekeeping genes β-actin and glyceraldehyde-3-phosphate dehydrogenase (GAPDH). RNA was extracted from HUVEC using the RNeasy mini kit (Qiagen) and quantified at 260/280 nm using a Nanodrop 2000 spectrophotometer (Thermo Scientific, UK). 1 μg of DNase-1-digested total RNA was reverse transcribed into cDNA using 1 μM oligo(dT) and Superscript reverse transcriptase (Invitrogen, Paisley, UK). cDNA was amplified in a 25 μl reaction solution containing 5 μl of cDNA template, 12.5 μL of iSYBR supermix, 0.5 pM sense and antisense gene-specific primers, and double distilled water (dd H_2_O). The cycling parameters were 5 min at 94 °C, 35 cycles at 95 °C for 30 sec, 58 °C for 30 sec, 72 °C for 30 sec. The primer sequences used are listed in Supplementary Table 1.

### Adenoviral infection

Adenoviruses encoding constitutively-active AMPK (AdCA-AMPK) (a gift from David Carling, Imperial College London) or green fluorescent protein (Ad-GFP) were amplified in HEK293A cells. Virus particles were purified according to manufacturers’ instructions using the AdEasyTM Virus Purification Kit (Stratagen, La Jolla, CA). Titrations were performed using the Adeno-XTM Rapid Titer Kit (Clontech Laboratories Inc., Mountain View, CA). Following optimisation experiments, HUVECs were infected at a multiplicity of infection (MOI) of 100 viral particles/cell in plain M199 at 37 °C for 2 h. The cells were then cultured for 24 h in M199/10% FCS and 7.5 μg/mL ECGF.

### CREB activation assay

A CREB (Phospho-Ser^133^) transcription factor assay kit (Cayman Chemicals, Cambridge, UK) was used to determine the transcriptional activation of CREB as per the manufacturers’ instructions. Nuclear extracts (10 μg) were added to a 96-well plate pre-coated with a specific double-stranded DNA (dsDNA) consensus sequence containing the cAMP response element (CRE) and incubated for 1 h at r/t. A wild-type oligonucleotide was used as a binding competitor. Unstimulated Hela nuclear extract was used as a positive control. The primary antibody was directed against phospho-CREB Ser^133^ and an HRP-conjugated secondary Ab was added to provide a sensitive colorimetric read-out at 450 nm

### Immunoblotting

EC were lysed in RIPA buffer (Sigma-Aldrich), containing a complete phosphatase and protease inhibitor (Roche, Burgess Hill, UK) at 4 °C for 15 minutes. 15 μg of protein was mixed with 7.5 μl of 4x NuPAGE® LDS Sample Buffer (Invitrogen) and 2 μl of DL-Dithiothreitol Solution (Sigma), and the sample boiled, prior to SDS-PAGE separation and transfer onto polyvinylidene difluoride membranes (Millipore, Massachusetts) using a semi-dry blotting apparatus (Bio-Rad). Membranes were blocked with 5% BSA and incubated with primary antibodies overnight at 4 °C. Following washing, membranes were incubated with a horseradish-peroxidase-conjugated secondary Ab diluted in 5% BSA, washed and developed using chemiluminescence ECL plus (GE Healthcare, UK) and exposed to Biomax light film (Kodak, Watford, UK). Densitometry of the protein bands was performed using ImageJ v1.45 Software (National Institute of Health, USA) and expressed relative to housekeeping proteins α-tubulin and GAPDH.

### Flow cytometry

HUVEC were harvested and resuspended in HBSS/1% FCS and stained with primary antibodies for 30 mins at 4 °C. The cells were washed, centrifuged and stained with FITC-labelled polyclonal anti-mouse IgG (DAKO, Stockport, UK) for 30 min at 4 °C. Following washing and resuspension in PBS, 10,000 cells were analysed using a CyAn™ ADP Analyzer (Beckman Coulter, Luton, UK) with Summit 4.3 software used to process data. Results are expressed as the relative fluorescent intensity, representing mean fluorescent intensity (MFI) with test monoclonal antibody divided by the MFI using the secondary antibody alone.

### Nuclear translocation studies

HUVECs were fixed in ice-cold methanol for 10 min, blocked with PBS/5% BSA and incubated at 4 °C overnight with rabbit anti-Nrf2 (1:200), rabbit anti-p65 (1:200) or negative control rabbit Ig fraction (DAKO), followed by washing and addition of either AlexaFluor goat anti-rabbit 488 (Invitrogen) or AlexaFluor donkey anti-rabbit 594 (Invitrogen) for 1 h at room temperature. Slides were washed, stained with Draq5 and mounted using Vectashield (Vector Laboratories, Peterborough, UK). Nuclear translocation was analysed using a Zeiss LSM META confocal microscope, with images obtained at 40× magnification from ten random fields of view. Fluorescence intensity of Nrf2 and p65 was quantified after correction for autofluoresence and defining threshold intensity from background fluorescence.

### Animals

Mice were housed in microisolator cages under controlled climactic conditions with autoclaved bedding. Irradiated food and drinking water were freely available. All animals were studied according to the guidelines from Directive 2010/63/EU of the European Parliament, with ethical approval from Imperial College London under UK Home Office Licence number PPL 70/7555. Female 8 week old C57BL/6 mice were fed for 48 hours on the standard laboratory diet (Harlan Teklad Global 18% Protein Rodent Diet, 2018) a modified celecoxib-supplemented form of the diet containing 1000 ppm celecoxib (Harlan) for 48 hours^32^. This dosing regimen achieves a plasma concentration of celecoxib in mice that is equivalent to the mean plasma concentration in humans following a single dose of celecoxib (1.53 µg/ml)^30,32^. Following treatment, mice were sacrificed by CO_2_ inhalation followed by cervical dislocation. Murine descending aorta was embedded vertically in OCT Embedding Matrix (CellPath Ltd, Newton, UK) and snap frozen in supercooled methylbutane (Sigma-Aldrich). 5 µm cryostat sections (Leica Microsystems, Wetzlar, Germany) were mounted on Superfrost microscope slides (Thermo Scientific, Waltham, MA), air-dried, fixed in ice-cold acetone for 5 minutes and stored at −80 °C.

### Prostaglandin E_2_ ELISA

The Prostaglandin E_2_ ELISA kit was from Arbor Assays (Ann Arbor, MI). 500 µl of blood was obtained from the sacrificed animals through cardiac puncture. The blood was left to coagulate for 2 h at 4 °C and then centrifuged at 14000 rpm for 15 mins. Serum was aspirated, re-centrifuged for 5 mins at 14000 rpm prior to storage at −80 °C. Samples were diluted 1:20 in assay buffer and analysed in duplicate according to the manufacturers’ instructions and read at 450 nm on a Synergy HT plate reader (Bio Tek Instrument, Swindon, UK).

### Immunohistochemistry

Transverse sections of frozen aorta were blocked for 1 h with 20% goat serum or 20% rabbit serum (DAKO) diluted in PBS. Polyclonal goat anti-mouse HO-1 or goat IgG isotype control (Santa Cruz) diluted 1:100 in PBS were added and incubated overnight at 4 °C. The process was repeated with polyclonal rabbit anti-mouse H-ferritin (Abcam, US) or rabbit IgG isotype control (Santa Cruz). Following washing, Alexa Fluor 568-conjugated rabbit anti-goat IgG or Alexa Fluor 546-conjugated goat anti-rabbit IgG (Invitrogen) (1:200) were added for 1 h in the dark. Slides were washed in PBS and rat anti-mouse CD31 mAb (Biolegend, SD, USA) was added as an endothelial marker and incubated for 2 h in the dark. Slides were washed and incubated with Alexa Fluor 488-conjugated goat anti-rat IgG and DAPI (4′6′-diamidino-2-phenylindole, Sigma) for 60 and 5 min respectively. Slides were mounted in Vectashield (Vector) and images captured with a Leica DM 2500 fluorescent microscope (Leica Microsystems, Wetzlar, Germany). Negative control area images were used in the calibration of green or red channel background intensity. Scan settings were set to optimise the signal/noise ratio for each emission wavelength. There was no detectable crossover between channels. Leica LAS EZ version 4.3 software was used to process the images which were quantified using ImageJ. Endothelial and adventitial areas were identified using the anti-CD31 stained co-localised image. Next, HO-1 and H-ferritin levels were quantified by recording the mean fluorescence intensity in the specific areas. The distribution of the pixel intensities in the red and green channels was computed using the histogram function. The background signal from the negative control images was subtracted.

### Statistical analysis

Data were analysed using GraphPad Prism Software (San Diego, CA, USA). Numerical data are presented as the mean of at least 3 individual experiments ± standard error (SEM). As indicated in the figure legends, differences between treatments were evaluated using the following methods: a one-sample t-test to compare two columns, an unpaired Student’s *t*-test, Mann-Whitney U test or, to evaluate three or more samples, the analysis of variance (ANOVA) was used. A Bonferroni correction was used to correct for multiple comparisons. *P* < 0.05 was considered significant.

## Electronic supplementary material

Supplementary material
