# Supplementary material for: Celecoxib exerts protective effects in the vascular endothelium via COX-2-independent activation of AMPK-CREB-Nrf2 signalling
Source: Sci Rep. 2018 Apr 19;8:6271. doi: 10.1038/s41598-018-24548-z (PMC5908847; doi:10.1038/s41598-018-24548-z)
Supplement: Supplementary file 1 — Supplementary material [file 41598_2018_24548_MOESM1_ESM.pdf]

Supplementary Material

Celecoxib exerts protective effects in the vascular endothelium via COX-2-independent activation of AMPK-CREB-Nrf2 signalling.

Fahad Al-Rashed[1,2], Damien Calay[1], Marie Lang[1], Clare C Thornton[1], Andrea Bauer[1], Allan Kiprianos[1], Dorian O Haskard[1], Anusha Seneviratne[1], Joseph J Boyle[1], Alex H Schönthal[3], Caroline P Wheeler-Jones[4], Justin C Mason[1*]

[1]Vascular Sciences and Rheumatology, Imperial Centre for Translational & Experimental Medicine, National Heart and Lung Institute, Imperial College London, London, UK, [2]King Fahad Cardiac Center, King Saud University, Riyadh, Saudia Arabia, [3]Department of Molecular Microbiology & Immunology, Keck School of Medicine, University of Southern California, Los Angeles, USA,[4]Comparative Biomedical Sciences, The Royal Veterinary College, London, UK.

Supplementary Table 1. qRT-PCR Primer sequences

Primer	Sequences
AMPKα1	Forward 5’-TCA GGA AGA TTG TAT GCA GGC CCA-3’<br>Reverse 5’-TTC ATG GGA ATC CAC CTG CAG GAT TA-3’
HO-1	Forward 5’-CAG TCT TCG CCC CTG TCT AC-3’<br>Reverse 5’-CTG GTG TGT AGG GGA TGA CC-3’
Nrf2	Forward 5’-TACTCCCAGGTTGCCCACA-3’<br>Reverse 5’-CATCTACAAACGGGAATGTCTGC-3’
NQO-1	Forward 5’-GGG CAA GTC CAT CCA ACT G-3’<br>Reverse 5’-GCA AGT CAG GGA AGC CTG CA-3’
Trx reductase-1	Forward 5’-CCA CTG GTG AAA GAC CAC GTT-3’<br>Reverse 5’-AGG AGA AAA GAT CAT CAC TGC TGA T-3’
H-ferritin	Forward 5’-CGACCGCGTCCACCTCG-3’<br>Reverse 5’-CTTTCATTATCACTGTCTGCC-3’
VCAM-1	Forward 5’-GGTGGGACACAAATAAGGGTTTTGG -3’<br>Reverse 5’ -CTTGCAATTCTTTTACAGCCTGCC -3’
β-Actin	Forward 5’-GAG CTA CGA GCT GCC TGA CG-3’<br>Reverse 5’-GTA GTT TCG TGG ATG CCA CAG GAC T-3’
GAPDH	Forward 5’-CAA CAG CCT CAA GAT CAT C-3’<br>Reverse 5’-GAG TCC TTC CAC GAT ACC-3’
IL-6	Forward: 5’-AAATGCCAGCCTGCTGACGAAC-3’<br>Reverse: 5’-AACAACAATCTGAGGTGCCCATGCTAC-3’

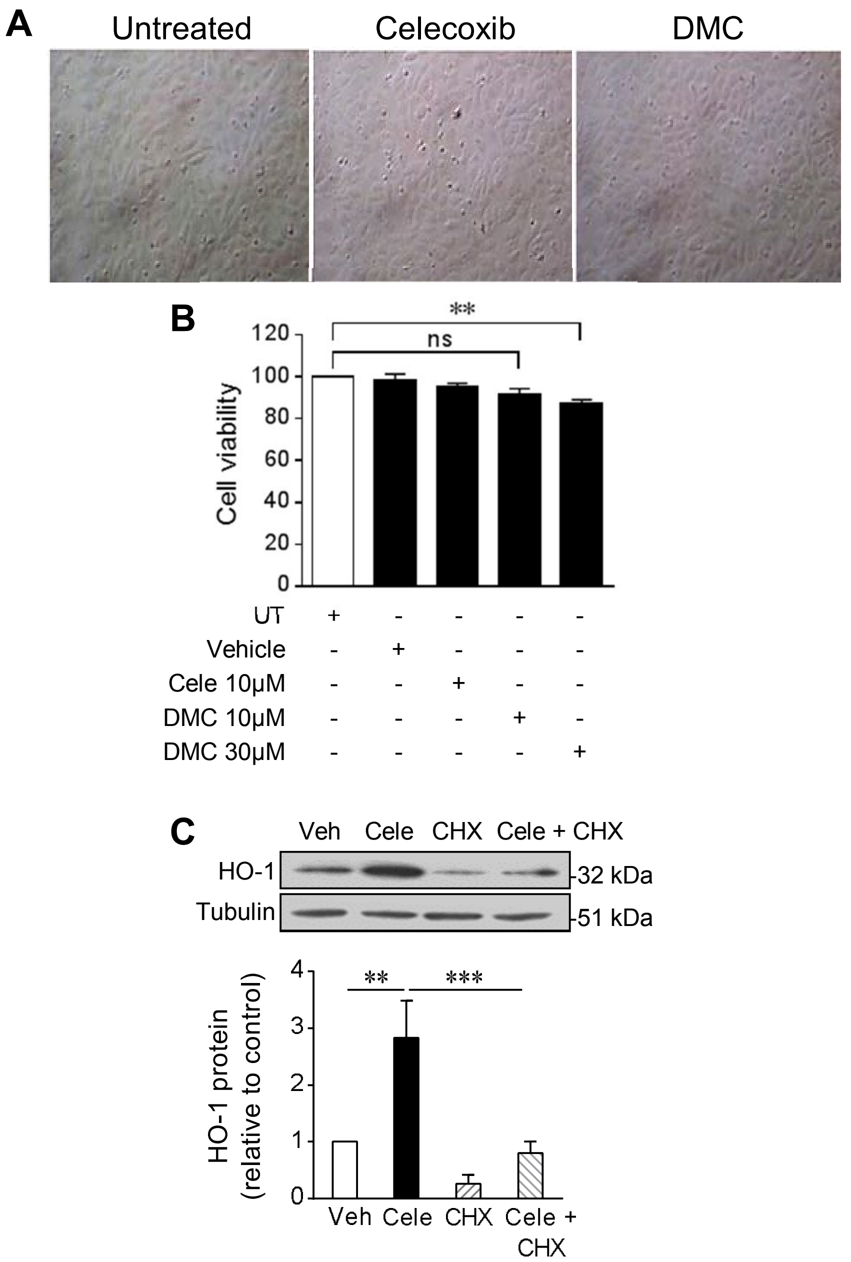

Supplementary Figure 1

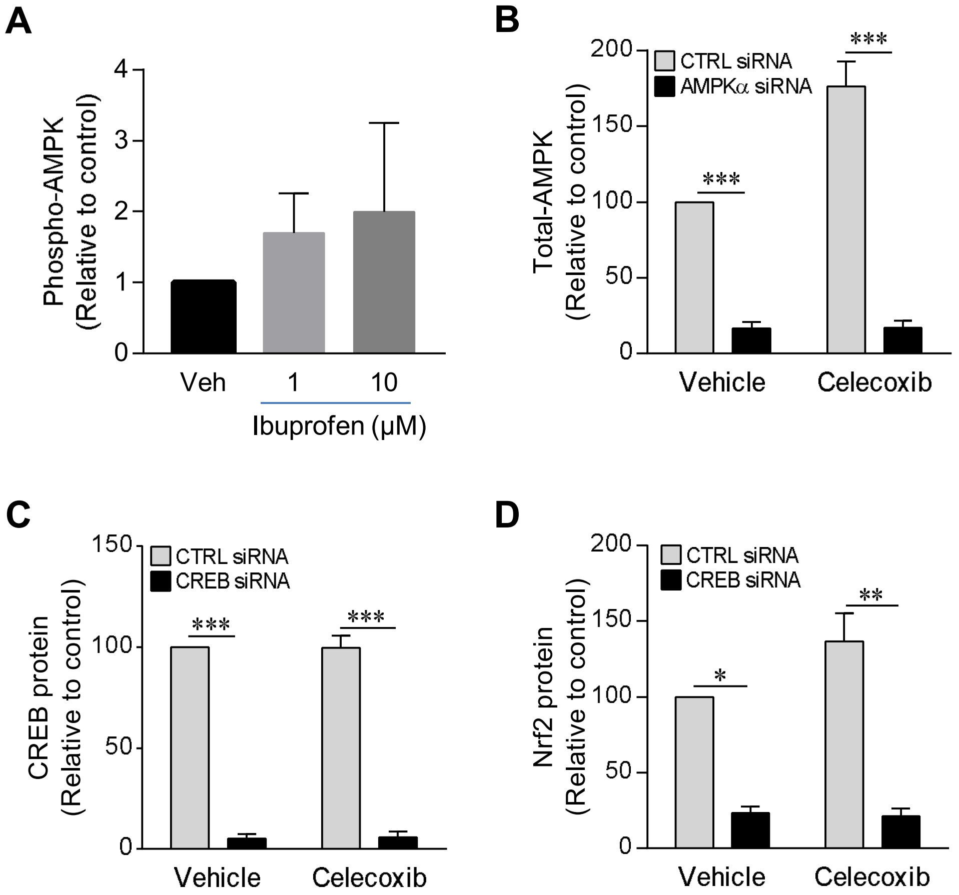

Supplementary Figure 2

A

	Fold increase in HO-1 mRNA (celecoxib relative to vehicle control)	Statistical analysis
H-Ferritin	4.17 ± 0.99	P<0.05
NQO1	1.19 ± 0.16	NS
Txnrd1	1.51 ± 0.17	P<0.05
Thioredoxin	1.04 ± 0.18	NS

NQO1: NAD(P)H dehydrogenase [quinone] 1; Txnrd1: Thioredoxin reductase 1

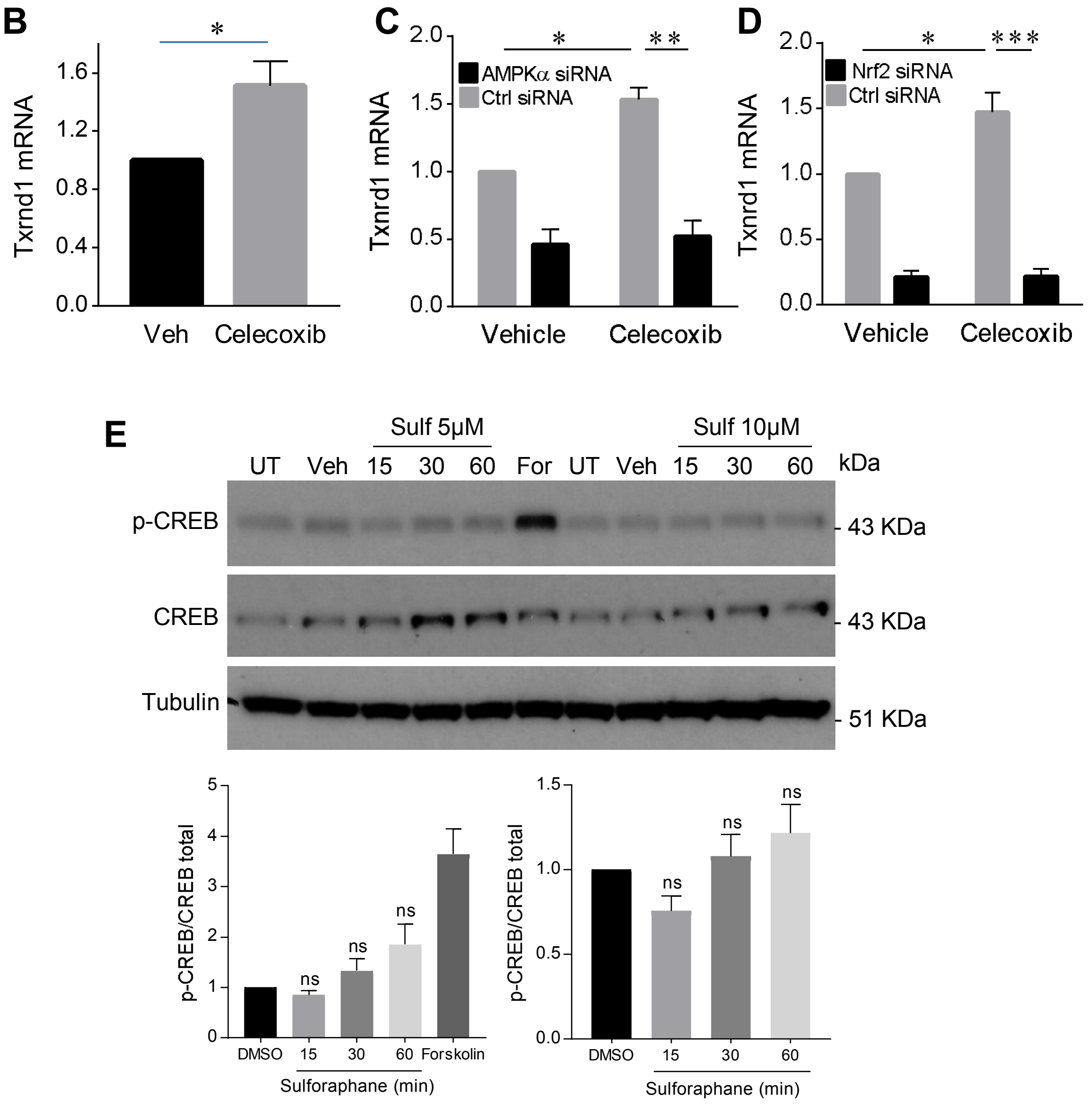

Supplementary Figure 3

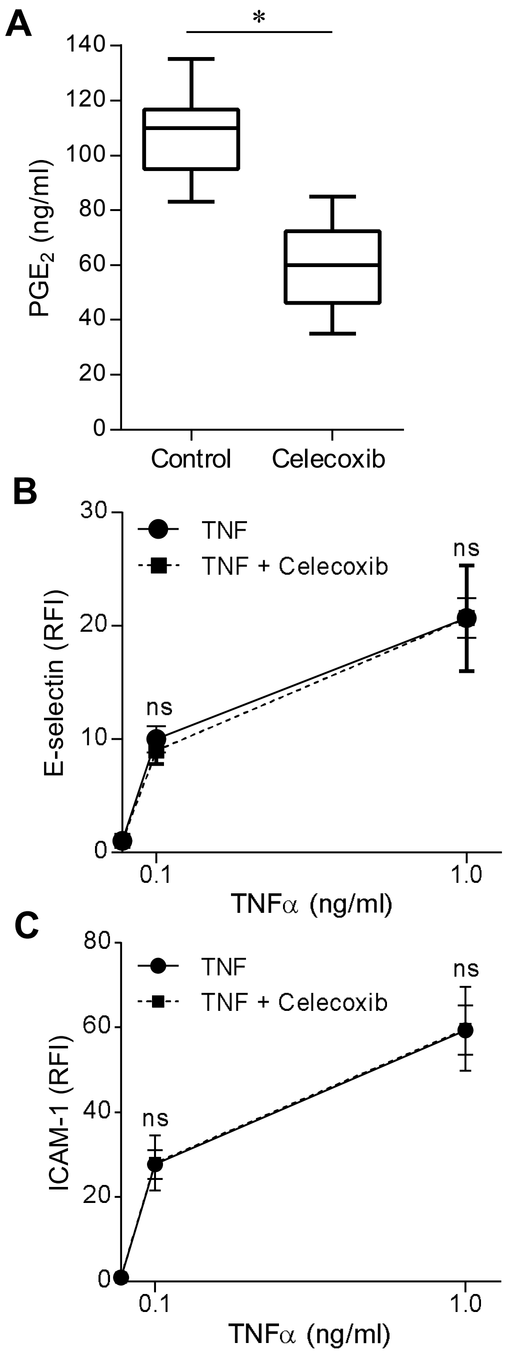

Supplementary Figure 4

Supplementary Figure 1. Lack of drug cytoxicity and role of protein synthesis in HO-1 induction. HUVEC monolayers were treated with celecoxib and DMC (10 µM) and cultured for 24 h prior to analysis by: (A) Phase-contrast light microscopy (x20 images) with no significant cellular toxicity observed, and (B) quantification of cell viability using a 3-(4,5 dimethylthiazol-2-yl)-2,5-diphenyltetrazolium bromide (MTT) assay. Data are presented as percent change in cell viability when compared with untreated cells (UT). (C) HUVECs were treated with celecoxib (10 µM) in the presence or absence of cycloheximide (CHX) 1µg/ml for 24 h, followed by analysis of HO-1 by immunoblotting and quantification by densitometry normalised corrected for α-tubulin and normalized to vehicle-treated cells.. Data in the figure are shown as mean ± SEM from 3 independent experiments, **P≤0.01, ***P≤0.001, using a one-way ANOVA with a Bonferonni correction.

Supplementary Figure 2. Ibuprofen treatment does not activate AMPK and validation of AMPK, CREB and Nrf2 siRNA oligonucleotides. HUVECs were treated with ibuprofen up to 10 µM and immunoblotted for phospho-AMPKα with bands quantified by densitometry normalised with respect to GAPDH and shown in a histogram incorporating date from 3 independent experiments. (B-D) HUVEC were left untransfected or transfected with (B) AMPKα siRNA, (C) CREB siRNA and (D) Nrf2 siRNA for 48 h prior to treatment with celecoxib for 24 h and immunoblotting for AMPKα, CREB and Nrf2 respectively. Bands were quantified by densitometry corrected for GAPDH, normalized to vehicle-treated cells and shown in histograms comprising data from 3 independent experiments. *P≤0.05, **P≤0.01, ***P≤0.001, using a two-way ANOVA.

Supplementary Figure 3. Celecoxib selectively induces Nrf2 target genes.
(A) and (B) HUVECs were treated with celecoxib (10 µM) or vehicle alone for 24 h prior to qRT-PCR analysis of H-ferritin, NQO1, Txnrd1 and thioredoxin mRNA. (C and D) HUVECs were left untransfected or transfected with: (C) AMPKα siRNA, (D) Nrf2 siRNA or control (CTRL) siRNA and Txnrd1 mRNA was analysed by qRT-PCR. (E). HUVECs were left untreated or treated with vehicle alone (Veh), sulforaphane (5 or 10 µM) for 15-60 mins, or forskolin (For) 60 µM for 20 mins followed by immunoblotting for phospho-CREB, CREB and tubulin. Data are normalized to vehicle-treated cells and shown as mean ± SEM, *P≤0.05, **P≤0.01, ***P≤0.001, using a two-way ANOVA or a one way ANOVA with correction for multiple comparisons .

Supplementary Figure 4. Celecoxib treatment of mice reduces serum PGE_2, but fails to inhibit E-selectin or ICAM-1 induction *in vitro.* (A) C57BL/6 female mice (n = 6 per group) were fed a diet containing 1000 ppm of celecoxib or a matched standard laboratory diet for 48 h. Following sacrifice serum was collected and PGE_2 quantified by ELISA. Results are expressed as the mean ± SEM and were analysed using a Mann-Whitney U test (*P ≤ 0.05). (B and C) HUVECs were pre-treated with vehicle or celecoxib (10 µM) for 24 h followed by TNF-α stimulation (up to 1 ng/ml) for (B) 6 h and (C) 16 h followed by flow-cytometric analysis of E-selectin and ICAM-1 respectively. Data are presented as relative fluorescence intensity (RFI), representing mean fluorescence intensity (MFI) with the antibody of interest divided by the MFI of the UT control (n = 4 experiments). NS = non-significant, using a two-way ANOVA.

Original immunoblots

Figure 1

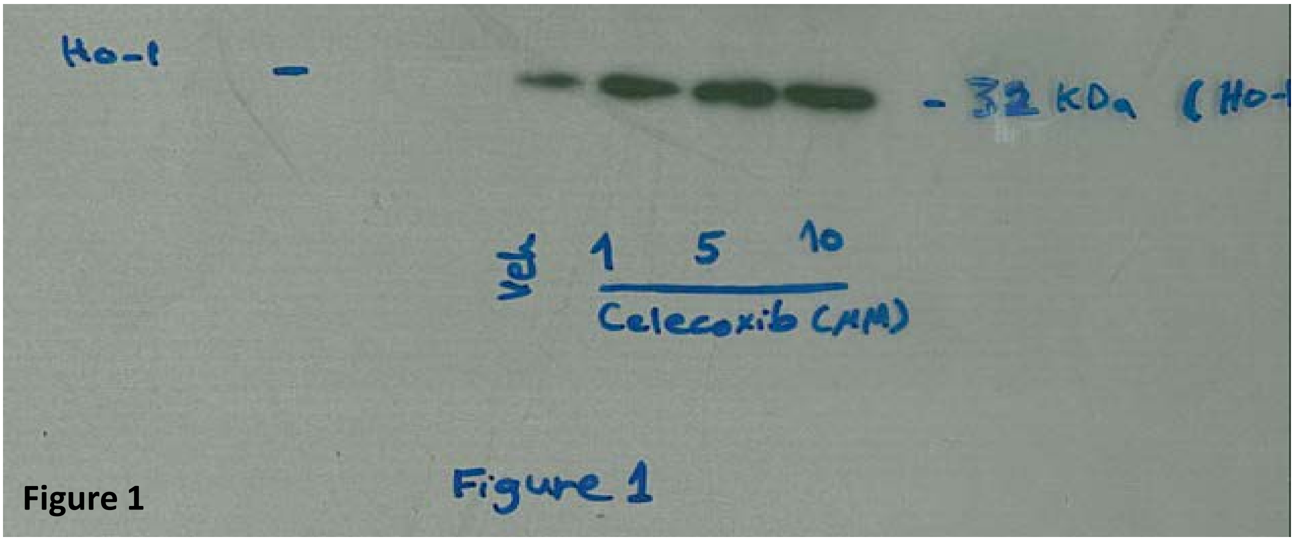

Figure 2B

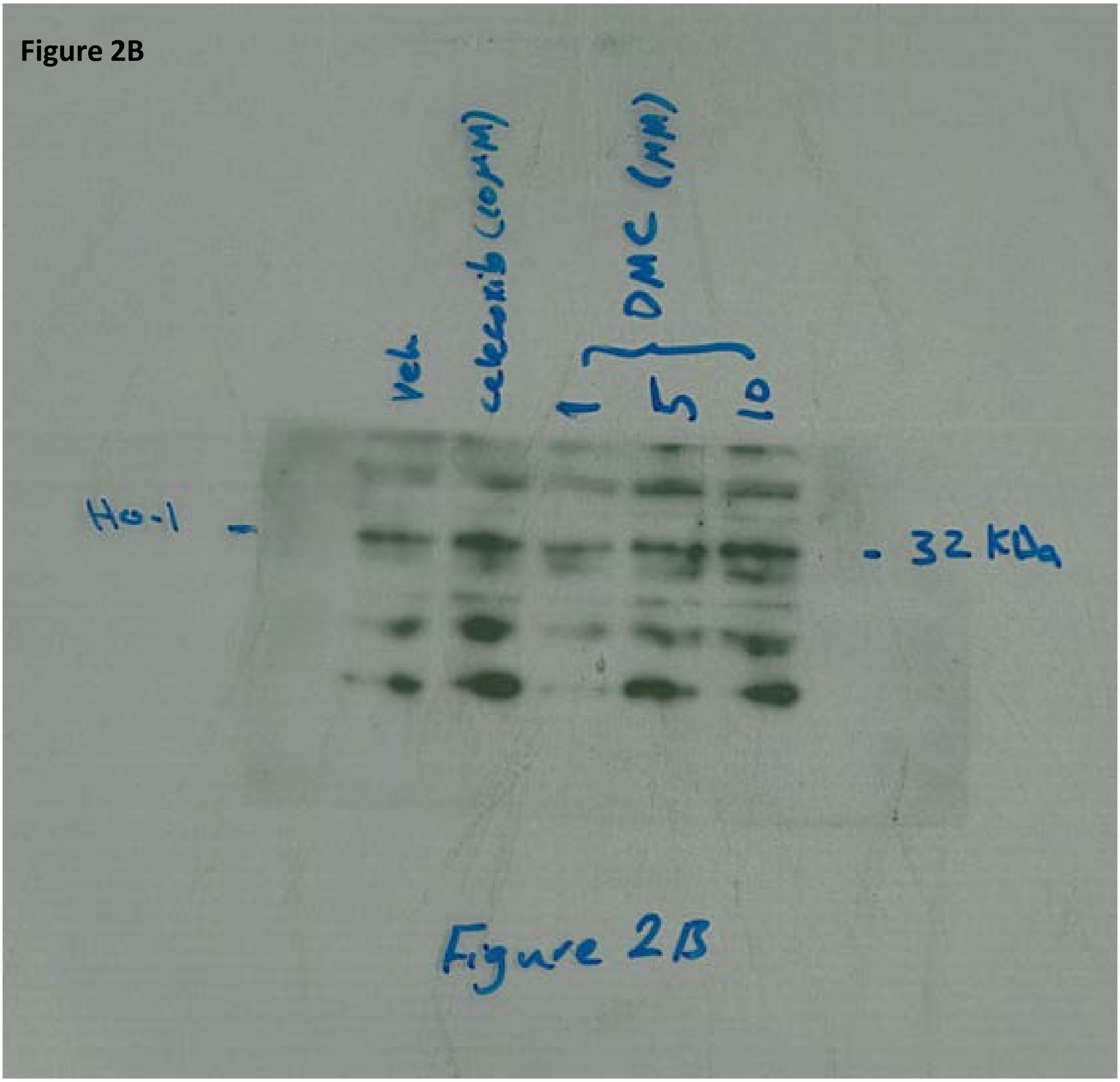

Figure 3C

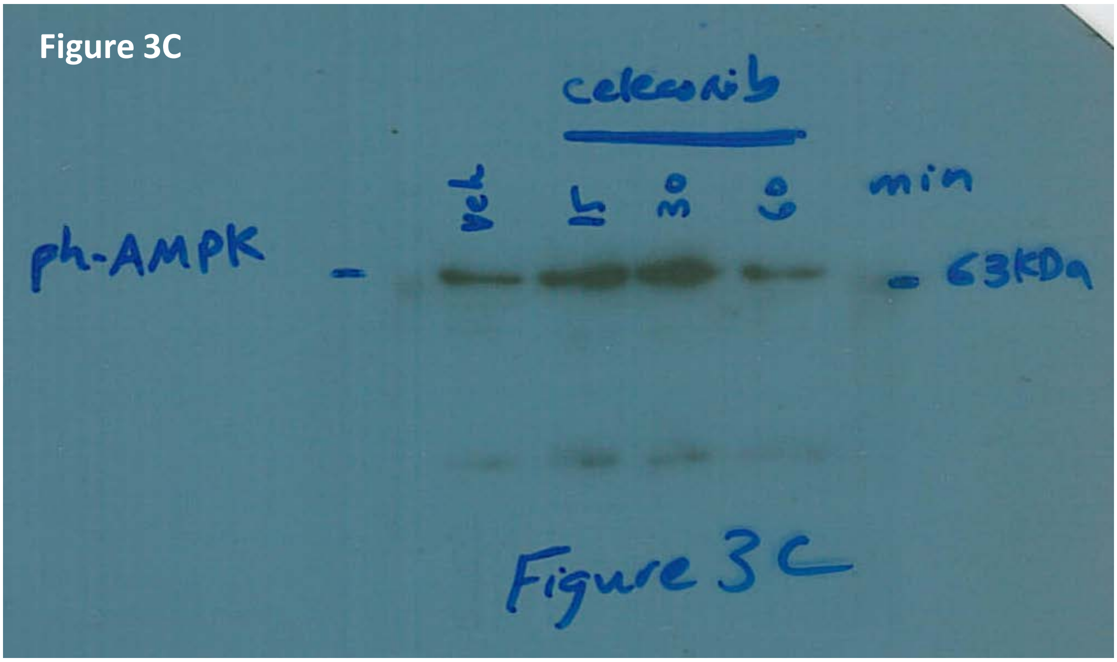

Figure 3F

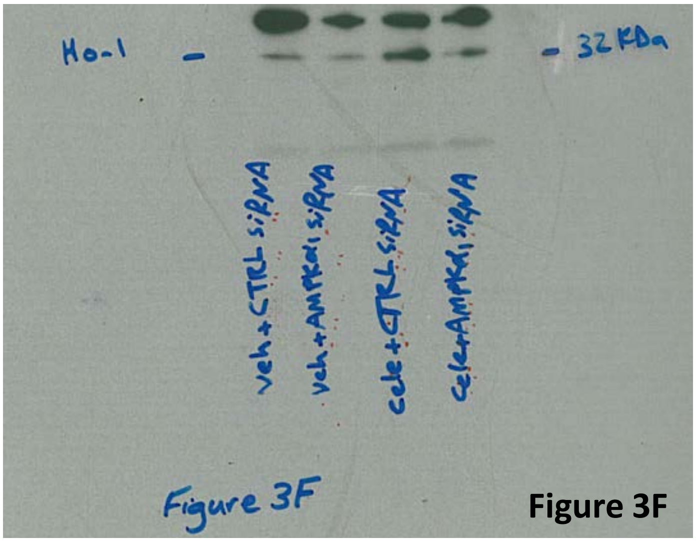

Figure 3Fii

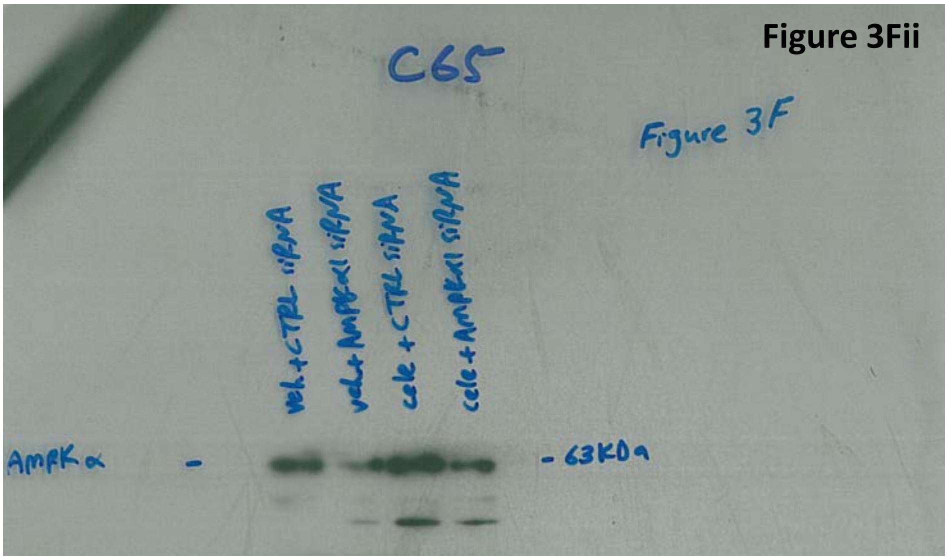

Figure 3D

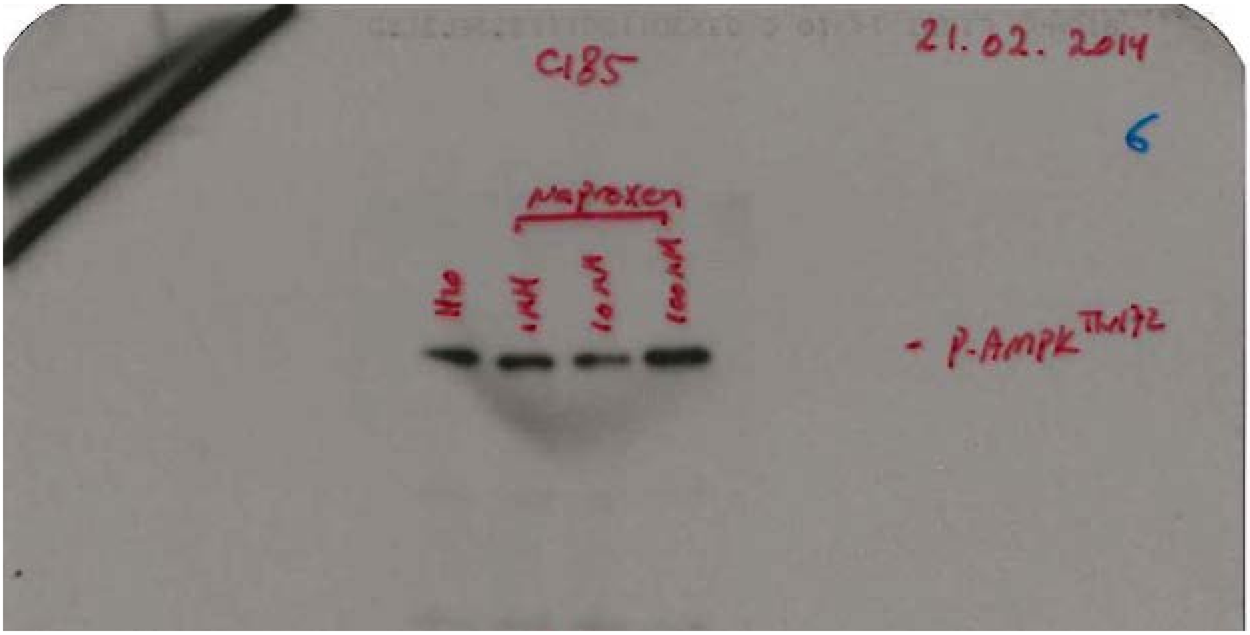

21.02.2014
C185
6
Naproxen
H2O
1mM
10mM
100mM
- p-AMPK

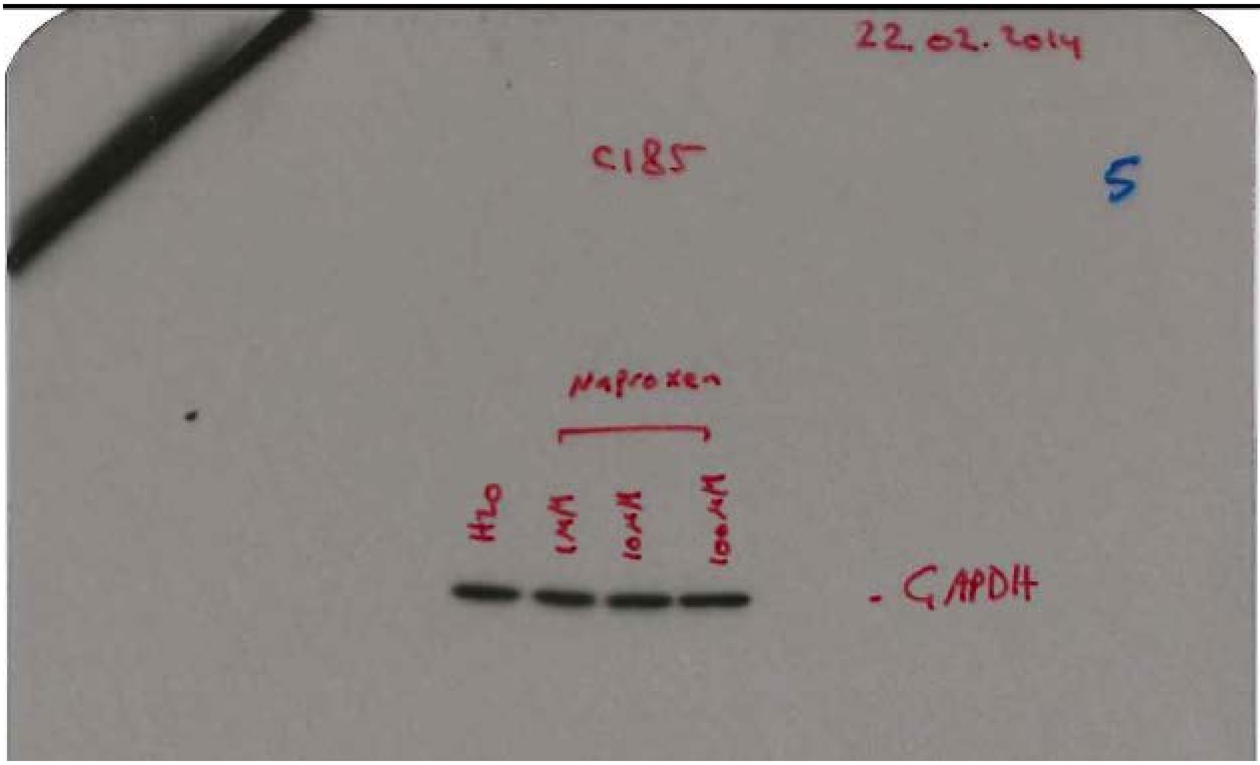

22.02.2014
C185
5
Naproxen
H2O
1mM
10mM
100mM
- GAPDH

Figure 4A

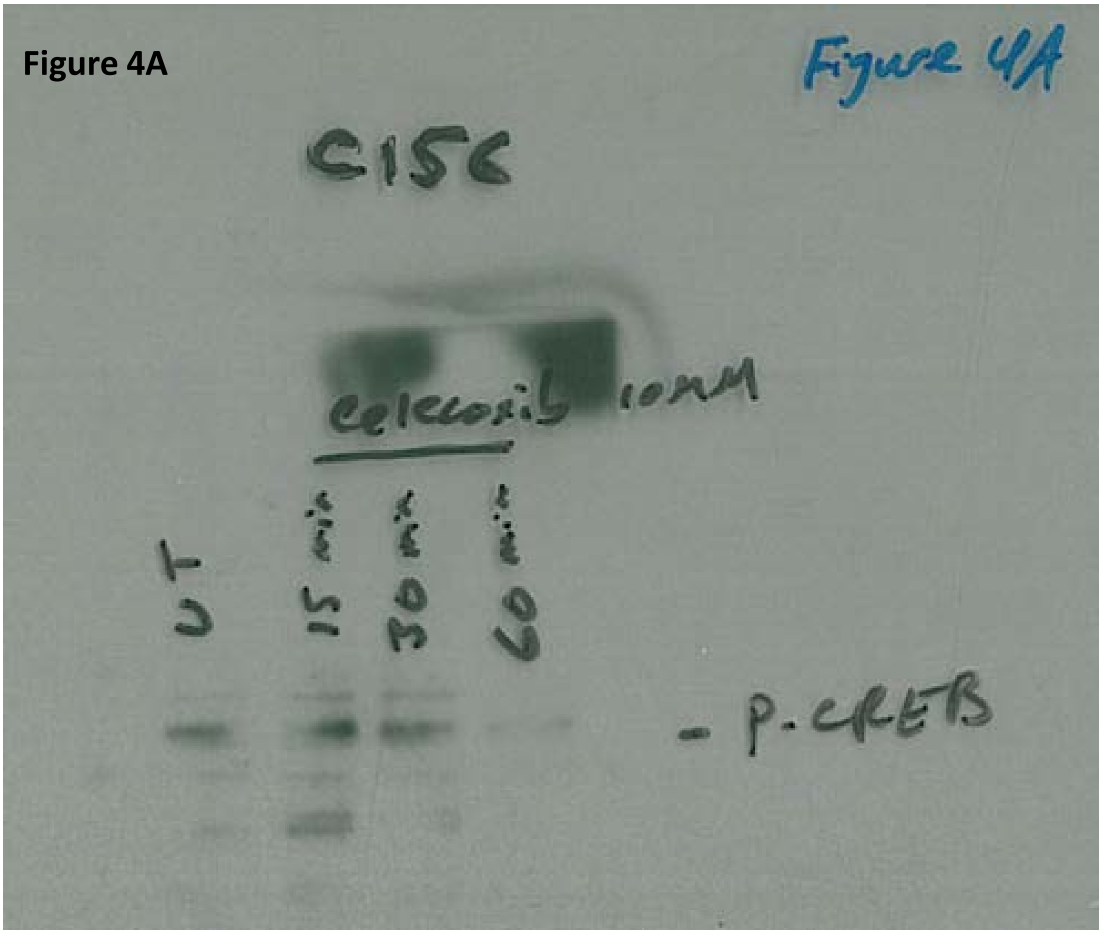

Figure 4C

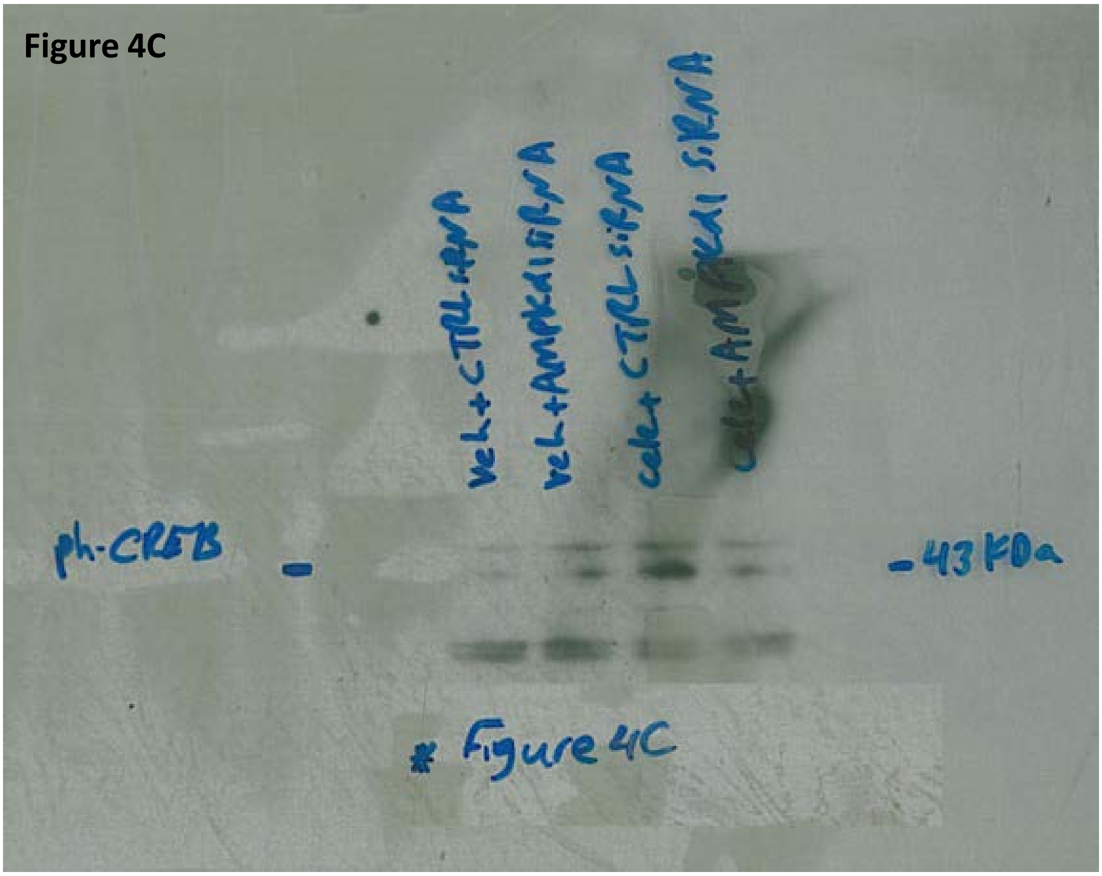

Figure 4E

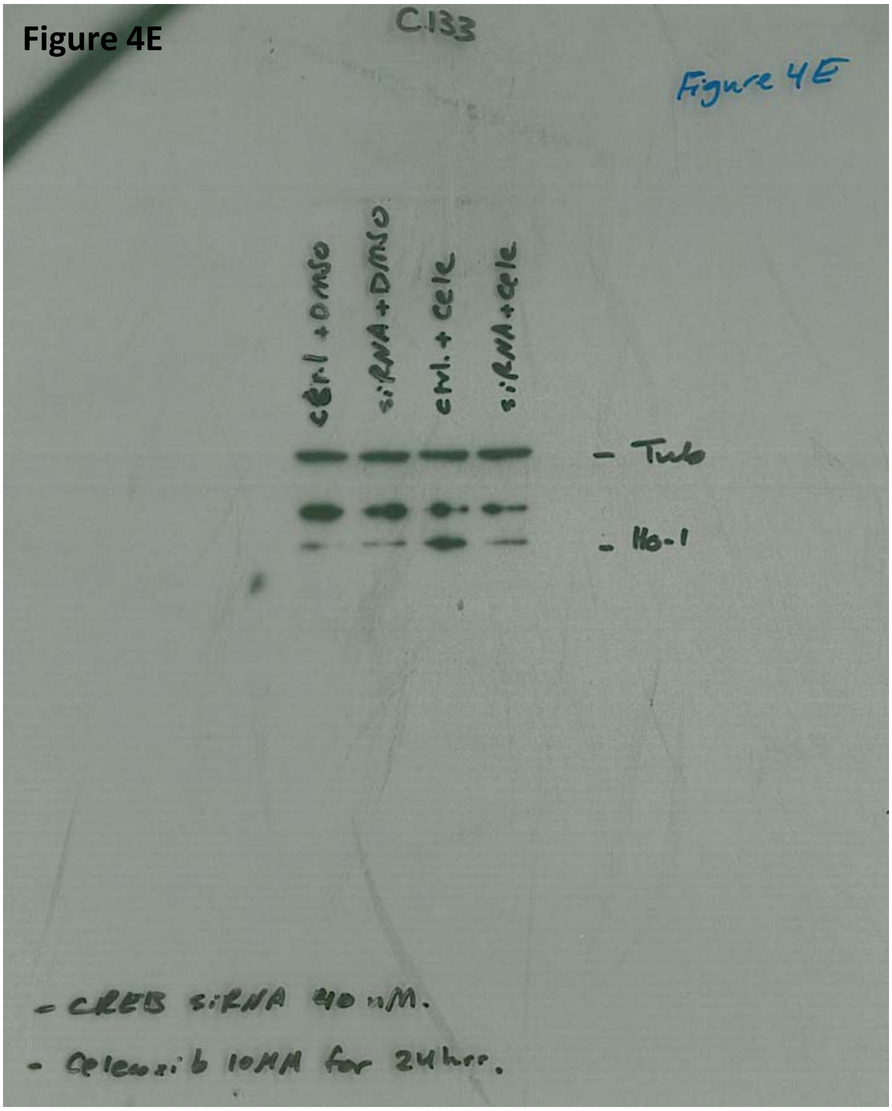
C133
Figure 4E
ctrl + DMSO
siRNA + DMSO
Ctrl. + Cele
siRNA + Cele
– Tub
– Ho-1
– CREB siRNA 40 nM.
– Celecoxib 10 μM for 24 hrs.

Figure 4F

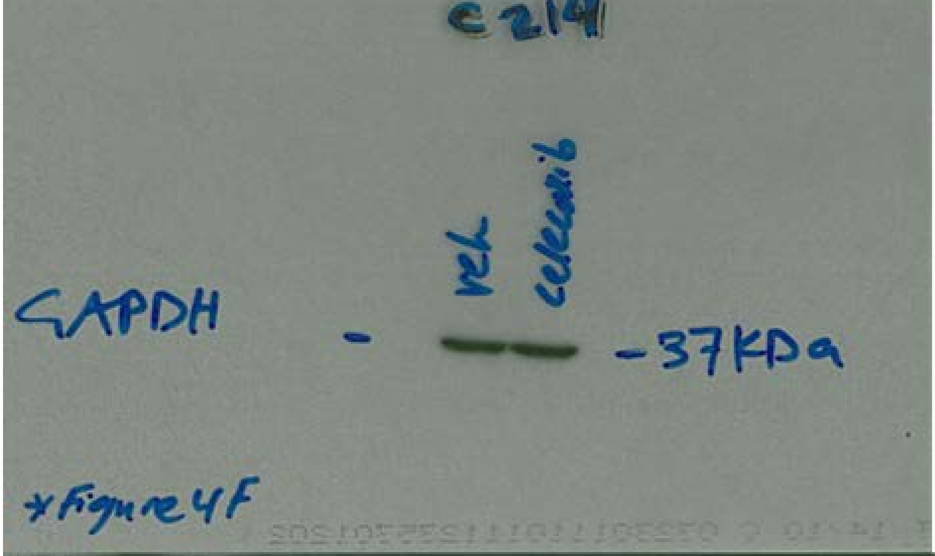

Figure 4F ii

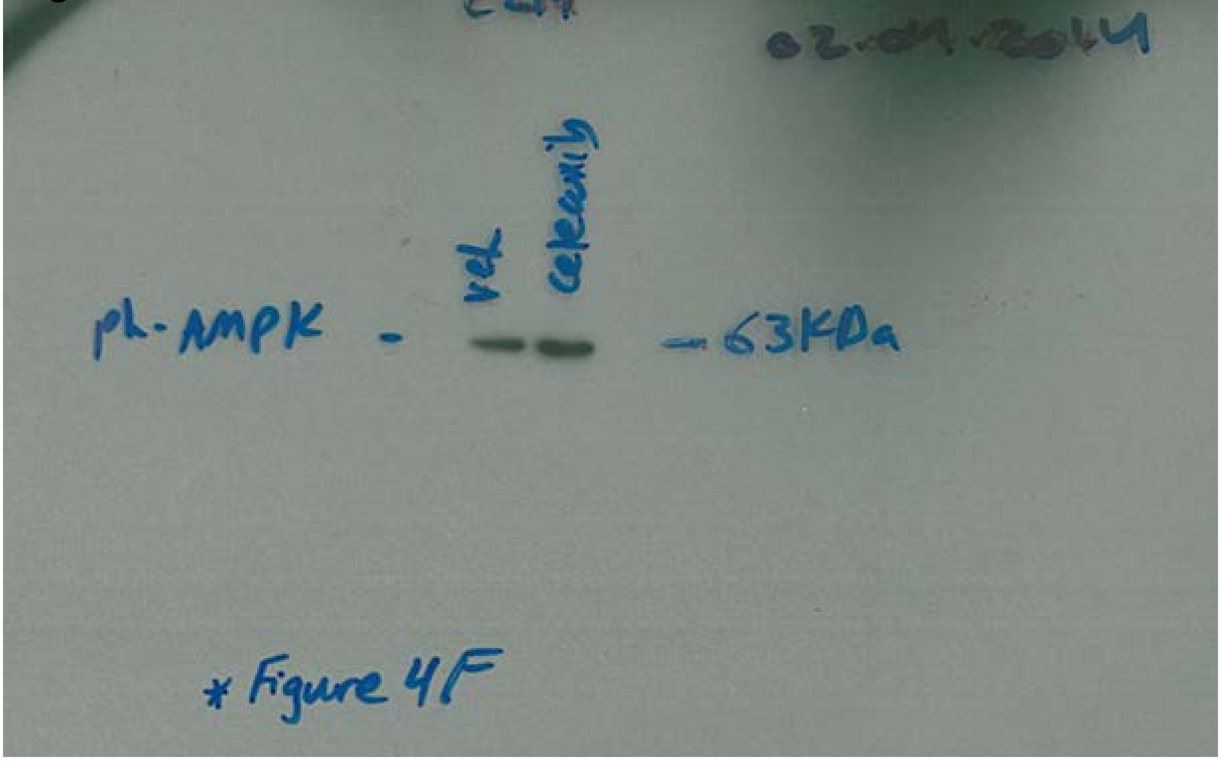

Figure 4F iii

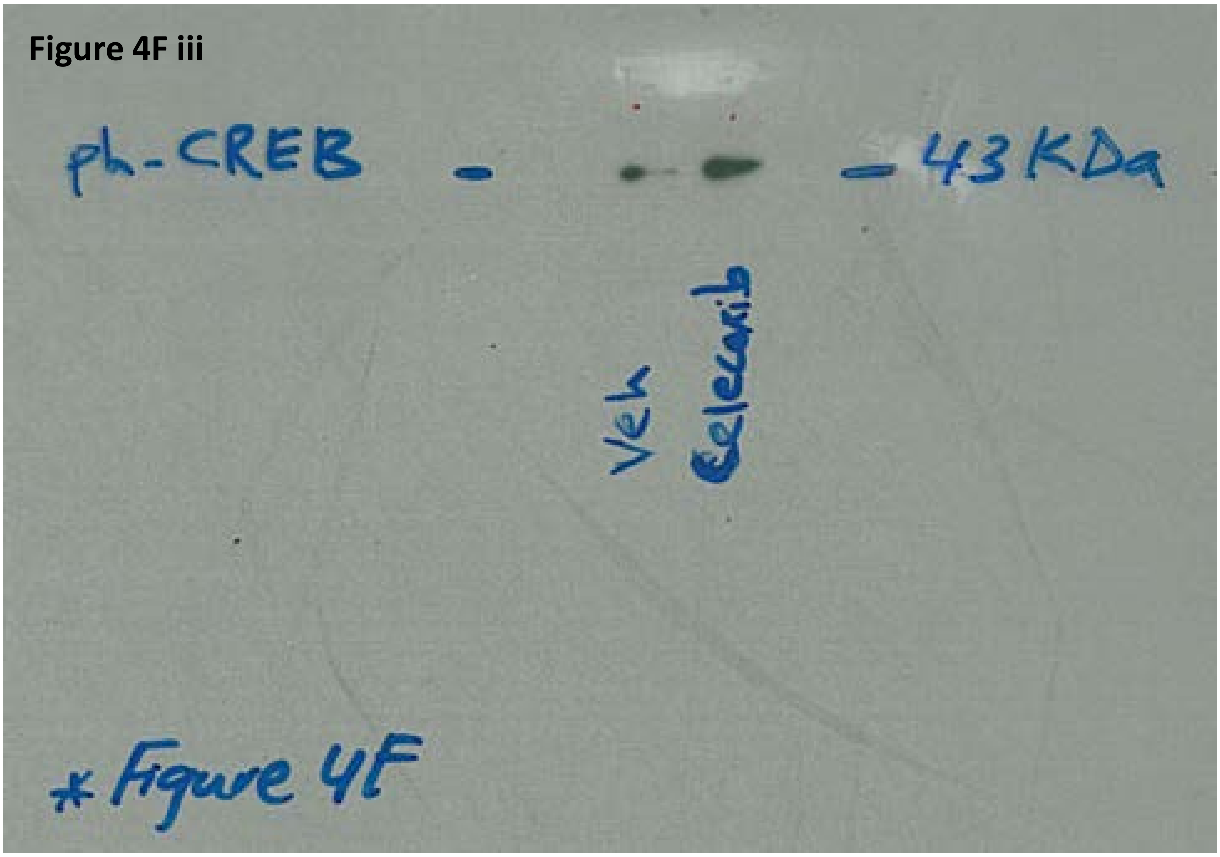

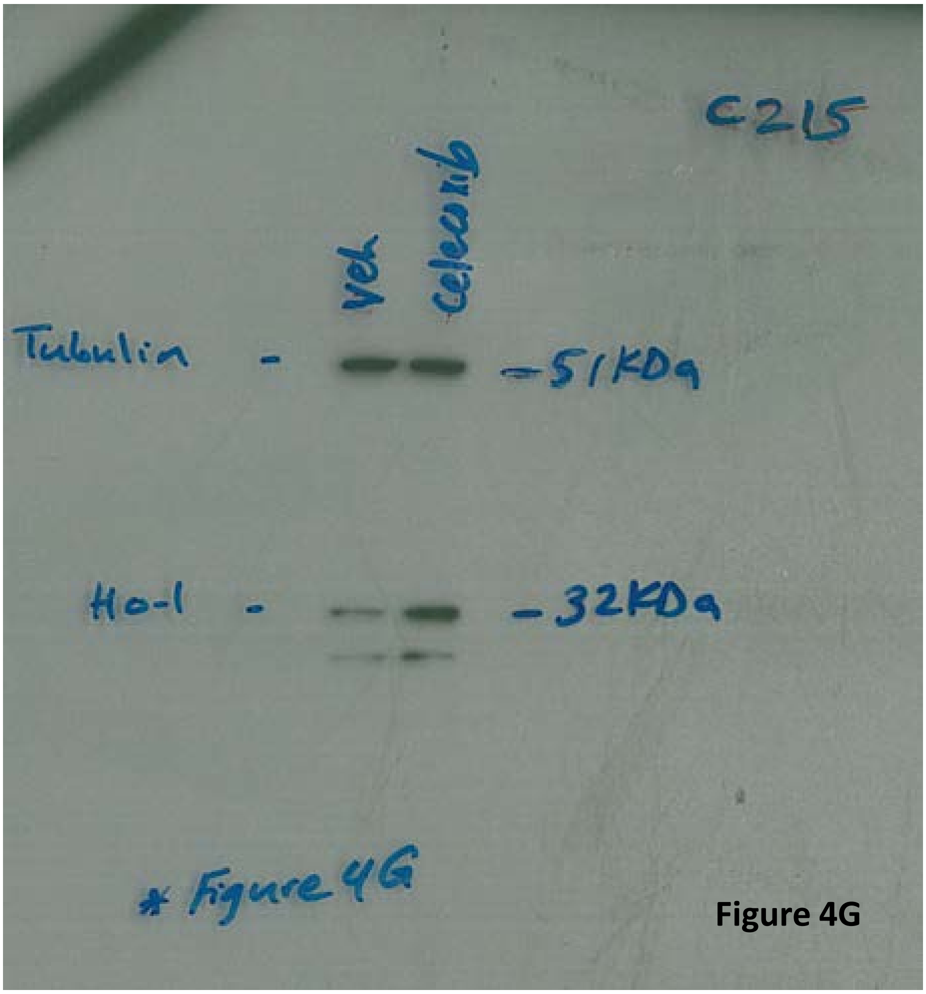

Figure 4G

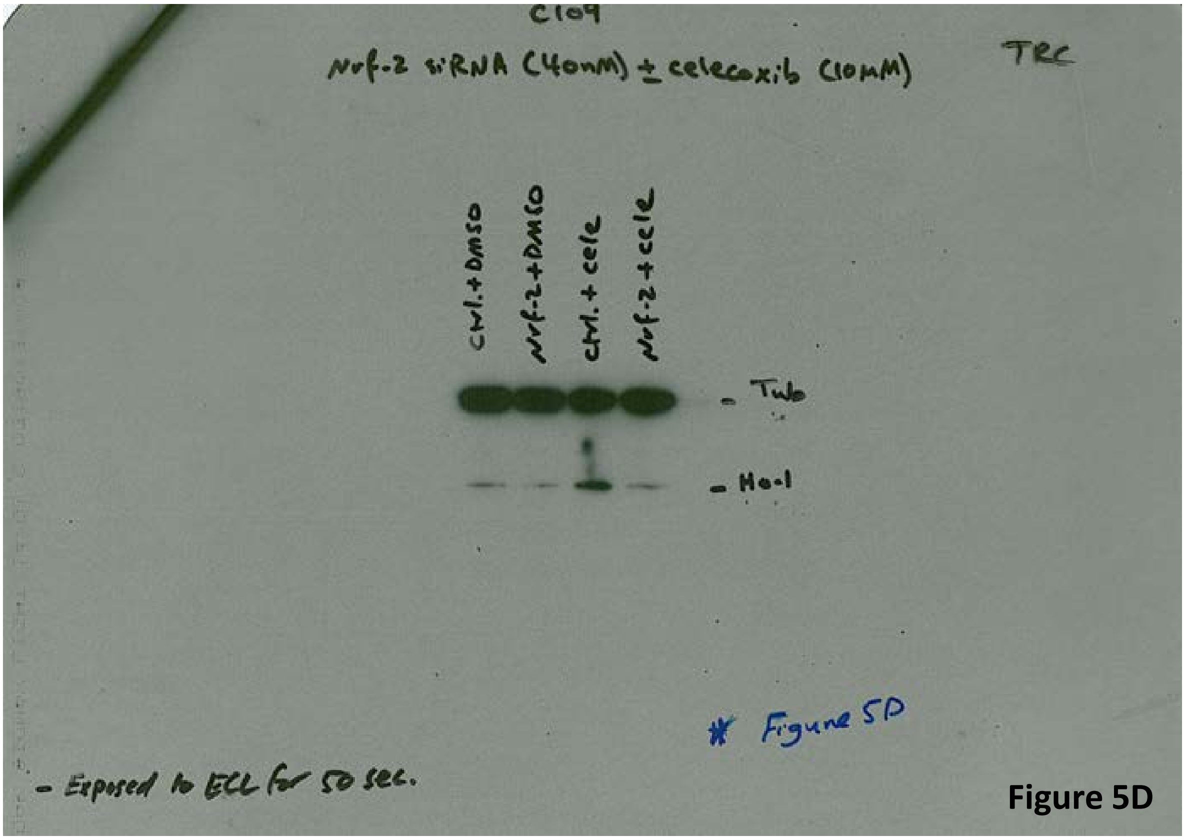

Figure 5D

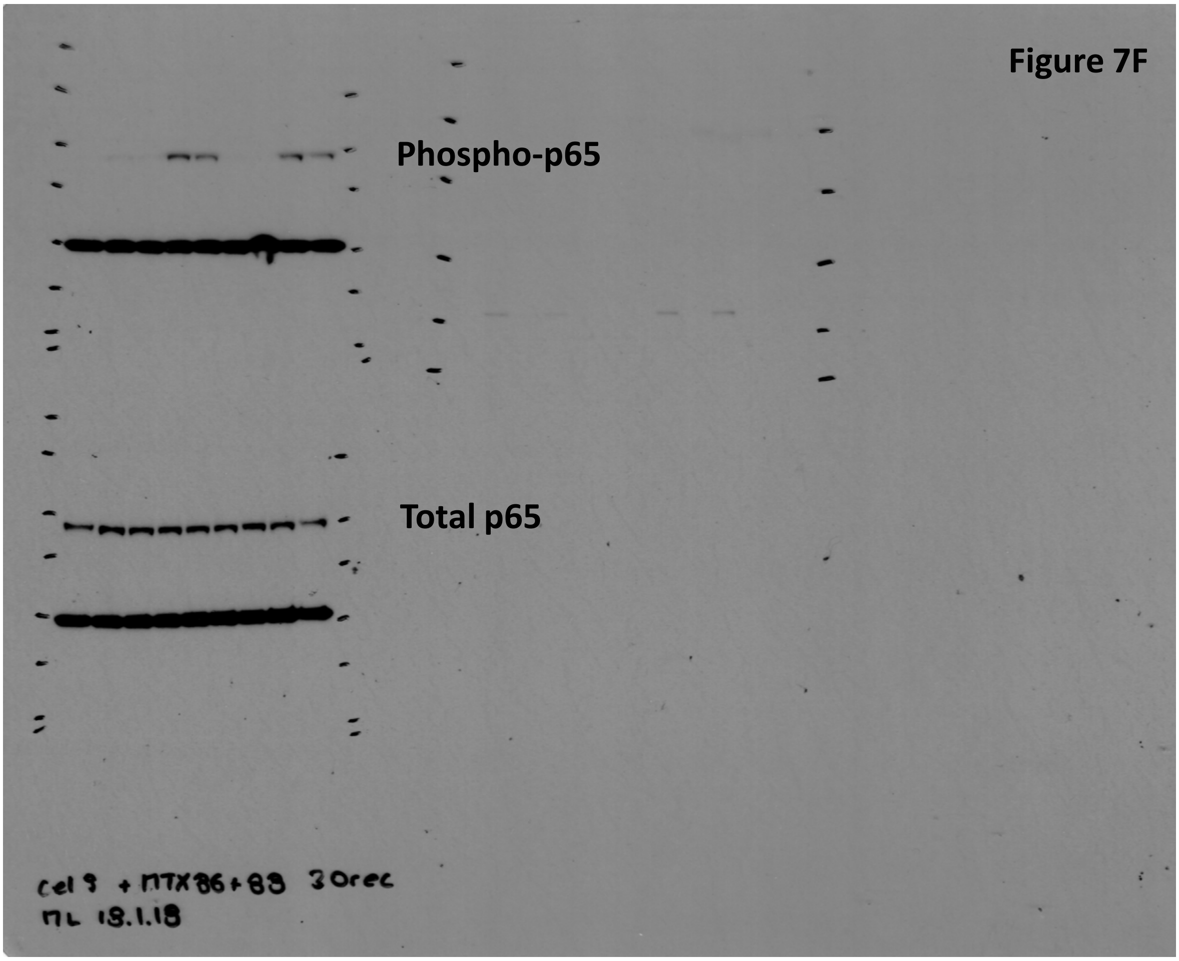

Figure 7F

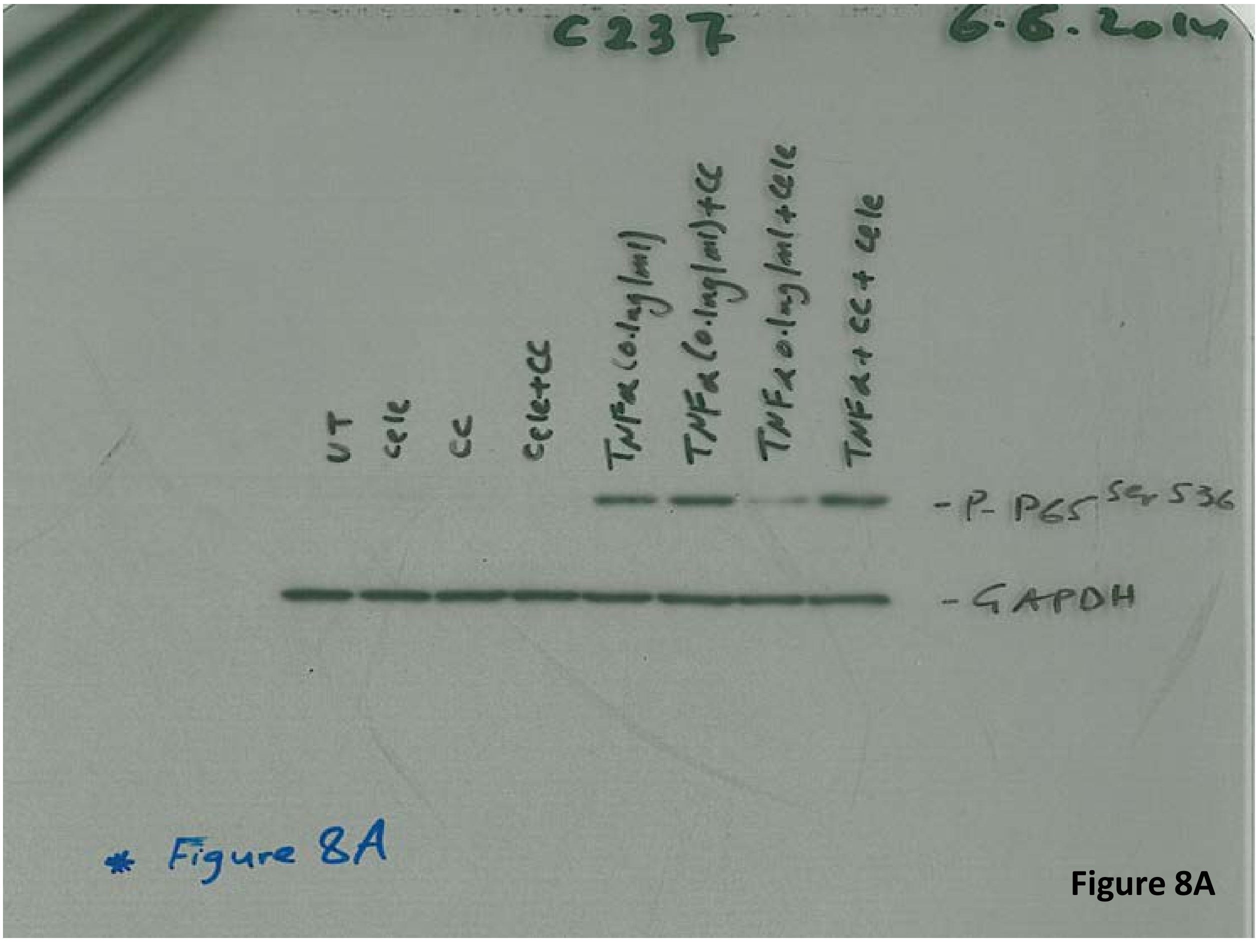
C237
6.6.2014
UT
cele
CC
cele+CC
TNFα (0.1ng/ml)
TNFα (0.1ng/ml)+CC
TNFα 0.1ng/ml + cele
TNFα + CC + cele
- P- P65 Ser 536
- GAPDH
* Figure 8A

Figure 8A

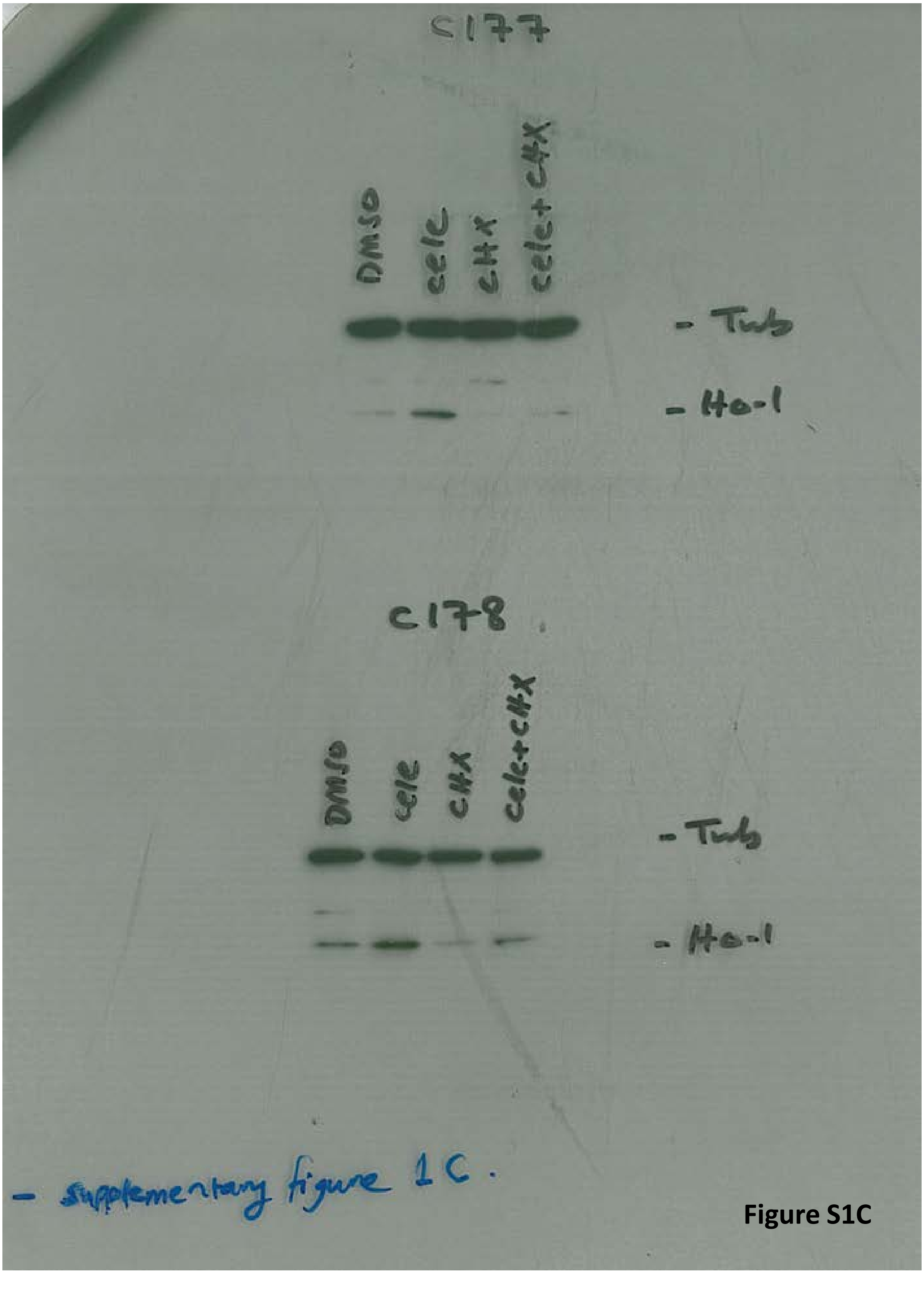
C177
DMSO
cele
CHX
cele+CHX
- Tub
- Ho-1
C178
DMSO
cele
CHX
cele+CHX
- Tub
- Ho-1
- supplementary figure 1C.

Figure S1C

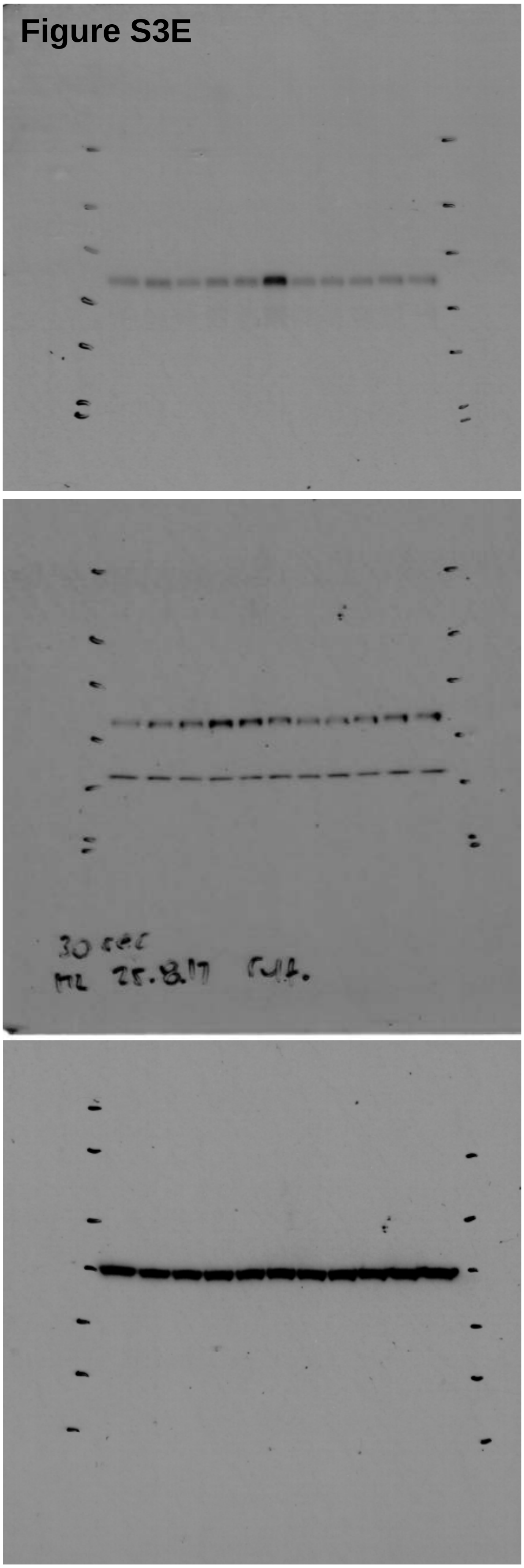

Phospho-CREB

Total CREB

Tubulin
